# Highly pathogenic avian influenza H5N1 clade 2.3.4.4b genotype B3.13 is highly virulent for mice, rapidly causing acute pulmonary and neurologic disease

**DOI:** 10.1038/s41467-025-60407-y

**Published:** 2025-07-01

**Authors:** Thomas Tipih, Vignesh Mariappan, Kwe C. Yinda, Kimberly Meade-White, Matthew Lewis, Atsushi Okumura, Natalie McCarthy, Ekaterina Altynova, Shanna S. Leventhal, Trenton Bushmaker, Chad S. Clancy, Emmie de Wit, Vincent J. Munster, Heinz Feldmann, Kyle Rosenke

**Affiliations:** 1 Laboratory of Virology, Division of Intramural Research, National Institute of Allergy and Infectious Diseases, National Institutes of Health, Hamilton, MT USA; 2 Rocky Mountain Veterinary Branch, Division of Intramural Research, National Institute of Allergy and Infectious Diseases, National Institutes of Health, Hamilton, MT USA

**Keywords:** Microbiology, Infection

## Abstract

The highly pathogenic avian influenza (HPAI) A(H5N1) clade 2.3.4.4b viruses, responsible for the current outbreak in dairy cows in the United States, pose a significant animal and public health threat. In this study, we compare disease progression and pathology of three recent clade 2.3.4.4b isolates derived from a cow, a mountain lion, and a mink to a human HPAI A(H5N1) isolate from Vietnam in mice. Inoculating C57BL/6J and BALB/c mice with all four HPAI A(H5N1) isolates results in comparable levels of virus replication in the lung inducing significant local pro-inflammatory cytokine responses and severe respiratory disease. Infecting C57BL/6J mice with the bovine isolate yields high viral titers in the brain, a significant pro-inflammatory cytokine response and neurologic disease. Our findings suggest the recent bovine isolate possesses enhanced neuroinvasive/neurovirulent disease causing fatal respiratory and neurologic disease in C57BL/6J mice.

## Abstract (web summary)

Intranasal inoculation of BALB/c and C57BL/6J mice with the bovine H5N1 clade 2.3.4.4b virus  results in rapid fatal disease characterized with high viral titers in lung and brain. Interestingly, only the C57BL/6J mice develop neurologic disease.

## Introduction

Since the emergence of the highly pathogenic avian influenza (HPAI) A(H5N1) (goose/Guangdong (gs/Gd) lineage) in China in 1996^1^, it has evolved into 10 main phylogenetic clades^2^. Clade 2 has been dominant and from 2005 HPAI A(H5N1) clade 2 viruses have disseminated across Asia, Europe, Africa, North and South America^3^. Since late 2021 H5N1 clade 2.3.4.4b viruses have continuously circulated in North America causing outbreaks in wild birds and domestic poultry with increasing spillover into wild mammals^4^. In March 2024, HPAI A(H5N1) clade 2.3.4.4b viruses were detected in dairy cows and unpasteurized milk in the United States^5^. Since the outbreak in dairy cows, 70 human cases of HPAI A(H5N1) have so far been reported in the United States caused by either the B3.13 genotype circulating in dairy cows or the D1.1 genotype circulating in birds^6–10^. Most human cases have thus far been mild, reporting mostly conjunctivitis although respiratory symptoms have been described^11–14^. In Canada however, a teenage girl required hospitalization after progression to respiratory failure but recovered after receiving combination antiviral therapy^15^.

Most cases in the US have been reported in persons working at dairy and poultry farms where cows or domesticated birds tested positive for HPAI A(H5N1) suggesting occupational exposure. However, the severe human cases reported in Wyoming and Louisiana following exposure to sick and dead birds in backyard flocks indicate that sources of exposures are not limited to commercial poultry and dairy operations. Despite evidence of mammal-to-mammal transmission^9,10^, the US Centers for Disease Control currently categorizes the public health risk for human infection as low^7^. The situation, however, could quickly change if the virus continues to evolve and acquire changes that may affect susceptibility, virulence, pathogenesis and transmission in mammalian species, specifically humans.

Prior to occurrence of HPAI A(H5N1) clade 2.3.4.4b infections in dairy cattle, outbreaks of HPAI A(H5N1) clade 2.3.4.4b viruses had been reported in mammals including farmed mink in Spain in 2022^16^, mink, arctic foxes and racoon dogs in Finland^17,18^, domestic cats in Poland^19^ and marine mammals in South America^20–22^. In February 2024, a mountain lion was found dead in Montana, USA, and a HPAI A(H5N1) clade 2.3.4.4b virus was isolated from lung tissue^23^. Similarly, natural infections in other wild terrestrial mammals have been reported^4,24–27^ altogether highlighting irregular avian-to-mammalian spillover leading to clinical infection of varying degree in several mammal species.

The current state around HPAI (A)H5N1 creates a vulnerable condition for animal and public health in the USA and worldwide. There are many gaps to fill including countermeasure development for which animal models are critical. In this study, we compare the virulence of A/Vietnam/1203/2004 (H5N1) (VN1203), a reference HPAI A(H5N1) clade 1 virus, to three recent HPAI A(H5N1) clade 2.3.4.4b virus isolates A/bovine/OH/B24OSU-342/2024 (bovine), A/mountain lion/MT/1/2024 (mountain lion), and A/mink/Spain/3691-8_22VIR10586-10/2022 (mink). The recent isolates are all the result of cross-species transmission likely from a wild bird source into mammalian species with the mink and bovine isolates causing significant mammal-to-mammal transmission^5,16^. We compare two potential exposure routes, nasal and orogastric, in C57BL/6J mice, an established model for influenza A viruses including HPAI H5N1^28^. We substantiate the initial findings in a different mouse strain (BALB/c). Finally, we characterize disease kinetics of the widely circulating bovine isolate in the two mouse strains. We show that nasal or orogastric inoculation of the bovine isolate results in a rapid disease progression in both BALB/c and C57BL/6J mice. Despite comparable viral tissue titers in the brains of both mouse strains, C57BL/6J mice are more prone to developing neurologic disease. These studies indicate the recent bovine isolate possesses enhanced neuroinvasive/neurovirulent disease resulting in both respiratory and neurologic disease in C57BL/6J mice.

## Results

### Sequence comparisons reveal close genetic relationship between bovine and mountain lion isolates

Amino acid sequence comparison of the bovine isolate with the corresponding sequences of the 3 additional isolates revealed the closest identity to the mountain lion (in most of the protein sequences) followed by the mink with the most distantly related being the VN1203 isolate (Supplementary Tables 1–9).

### Bovine isolate rapidly induces fatal disease course

**Fig. 1:**
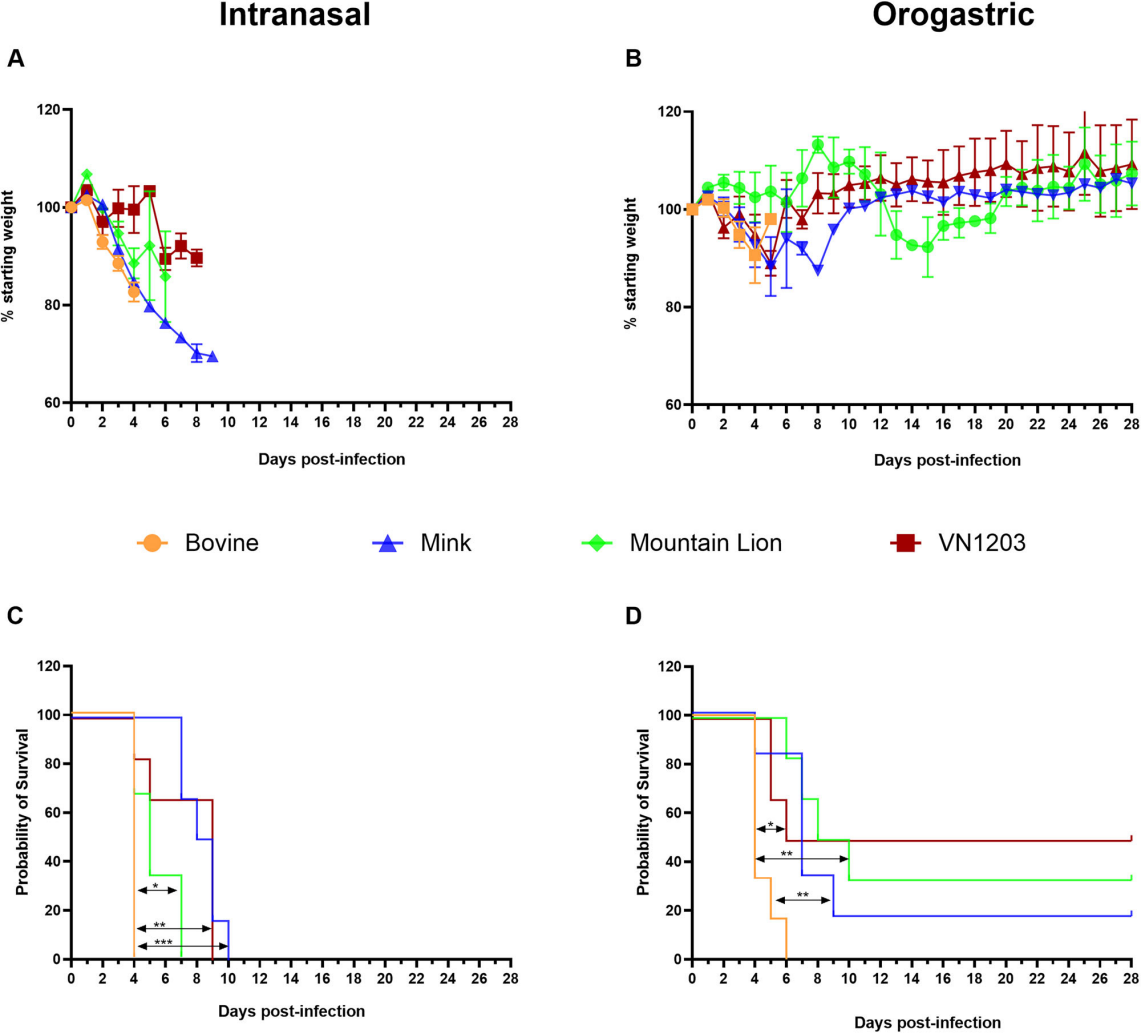
Disease progression following HPAI A(H5N1) infection. Six-week-old C57BL/6J mice (*n* = 10 per group) were inoculated via either intranasal or orogastric routes with 10^5^ TCID_50_ of either A/bovine/OH/B24OSU-342/2024, A/mountain lion/MT/1/2024), A/mink/Spain/3691-8_22VIR10586-10/2022, or A/Vietnam/1203/04. Mice (*n* = 6) in each group were monitored for clinical signs and survival. **A** Weight loss following intranasal inoculation. **B** Weight loss following orogastric inoculation. **C** Survival following intranasal inoculation. Survival proportions were calculated using the log-rank test with Bonferroni correction for multiple comparisons. ns *p* > 0.05, *p < 0.05, **p < 0.01, ****p < 0.0001. **D** Survival following orogastric inoculation. Note, animal group sizes change over time due to animals succumbing to infection. **A**, **B** Data shown as mean plus standard error of the mean.

To compare virulence and pathology of the recent bovine, mink and mountain lion isolates to the reference VN1203 isolate, groups of C57BL/6J mice (*n* = 10) were inoculated via intranasal or orogastric routes with 10^5^ TCID_50_ of each isolate. Four animals per group were euthanized at day 4 post inoculation (PI) for virus titer determination and histopathology. Six animals per group were monitored daily for clinical signs and survival until day 28 PI. All virus isolates induced weight loss (Fig. 1A, B); additional disease signs included ruffled fur and hunched posture followed by hypoactivity and rapid/labored breathing. Neurological signs, however, comprising of tremors, ataxia, hyperactivity, and circling were observed in all C57BL/6J mice inoculated with the bovine isolate. Two animals inoculated with the mountain lion isolate (1 each intranasally and orogastric) also exhibited minor neurologic signs (hyperactivity). Inoculation of mice with the bovine isolate by either route resulted in uniform lethality with intranasally inoculated mice succumbing by day 4 and those orogastric inoculated by day 6 PI (Fig. 1C, D). Intranasal inoculation with the mink, mountain lion and reference VN1203 isolates also resulted in uniformly lethal disease. Disease progression was significantly extended reaching endpoint criteria between 4–10 days PI when compared to infection with the bovine isolate (Fig. 1C). Orogastric inoculation with the VN1203 isolate caused disease but was not uniformly lethal and similar results were found with the mink and mountain lion isolates (20–50% probability of survival) (Fig. 1D). Survival rates were significantly lower for the bovine isolate when compared to the mink, mountain lion and VN1203 isolates (Fig. 1C, D). Overall, the results show that the intranasal route resulted in more rapid and severe disease than orogastric inoculation and that the bovine isolate was most virulent resulting in additional neurologic disease.

### Bovine isolate replicated to high titers in lung and brain tissue

**Fig. 2:**
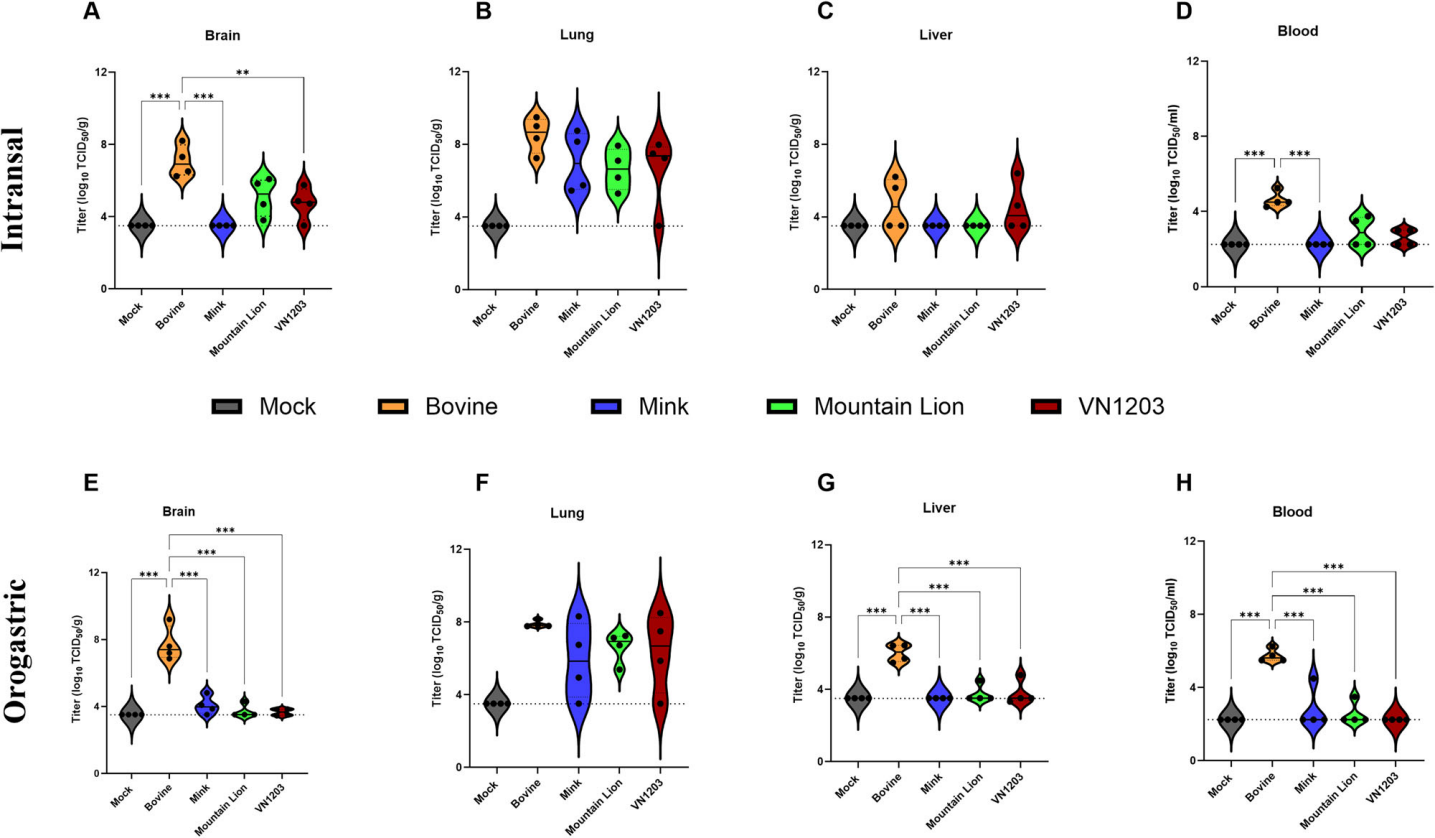
Infectious virus titers in organs following HPAI A(H5N1) infection. Six-week-old C57BL/6J mice were inoculated with 10^5^ TCID_50_ of A/bovine/OH/B24OSU-342/2024, A/mountain lion/MT/1/2024), A/mink/Spain/3691-8_22VIR10586-10/2022, or A/Vietnam/1203/2004. On day 4 post inoculation, 4 animals from each group were euthanized to determine virus titers in organs (lung, liver, brain) and blood. **A**–**D** Intranasally inoculated mice. **E**–**H** Orogastric inoculated mice. Dashed line indicates limit of detection. Statistical analyses were performed using one-way ANOVA with Tukey’s multiple comparison. ns *p* > 0.05, *p < 0.05, **p < 0.01, ****p < 0.0001. Comparisons with *p* values > 0.05 were not displayed. Violin plots show interquartile range, median and density curve.

To determine viral replication in organ tissues of Dulbecco’s Modified Eagle Medium (DMEM) inoculated (mock) or HPAI A(H5N1) inoculated C57BL/6J mice, 4 animals from each group were euthanized on day 4 PI at peak disease. Amongst the sampled tissues, virus titers were highest in the lung samples and lowest in liver and blood samples for all the virus isolates (Fig. 2A–H). Consistent with neurological signs observed in C57BL/6J mice, high virus titers were found in the brains of animals infected with the bovine isolate compared to those from inoculations with the mink, mountain lion and VN1203 isolates which either were negative or low (Fig. 2A, E). Significantly higher viremia was detected in mice inoculated with the bovine isolate compared to those inoculated with the mink, mountain lion and VN1203 isolates of which most of the mice were negative (Fig. 2D, H). No significant differences in organ virus titers were observed between the orogastric and nasal routes. Overall, the bovine isolate demonstrated enhanced replication in organs, caused systemic infection and displayed neurotropism. In contrast, most of the mice inoculated with the mink, mountain lion, and VN1203 isolates manifested limited evidence of systemic infection.

### Bovine isolate caused neurologic disease in the absence of brain lesions

**Fig. 3:**
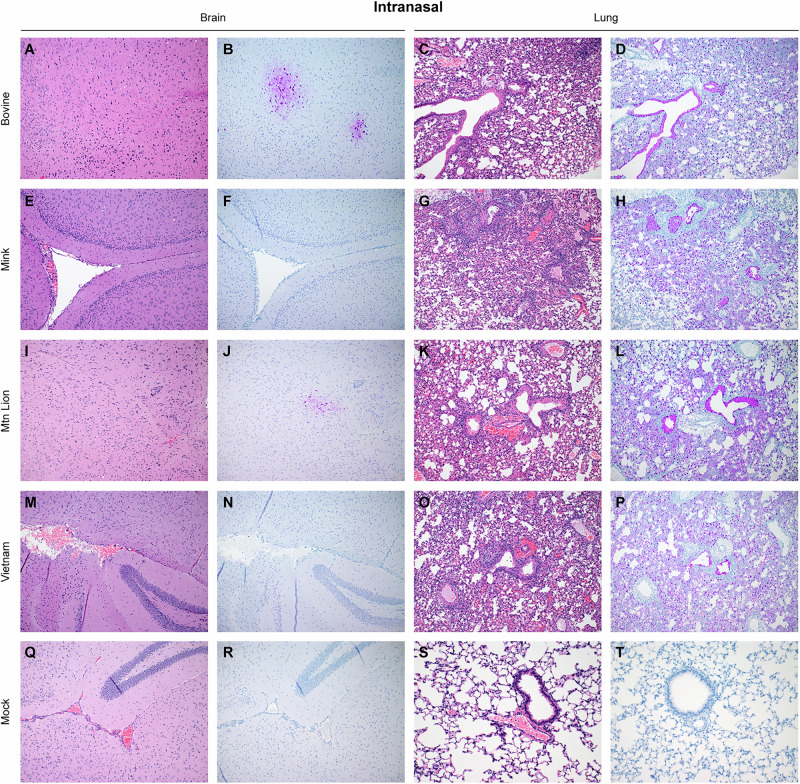
Histologic lesions and antigen expression following HPAI A(H5N1) intranasal inoculation. Six-week-old C57BL/6J mice were inoculated intranasally with 10^5^ TCID_50_ of A/bovine/OH/B24OSU-342/2024, A/mountain lion/MT/1/2024), A/mink/Spain/3691-8_22VIR10586-10/2022, or A/Vietnam/1203/2004. A subset of animals (*n* = 4) was euthanized at day 4 post inoculation for histopathologic evaluation. Representative sections from each group shown. **A**, **E**, **I**, **M**, **Q** Hematoxylin & eosin stain of brain sections. **C**, **G**, **K**, **O**, **S** Hematoxylin & eosin stain of lung sections. **B**, **F**, **J**, **N**, **R** Immunohistochemistry staining of brain sections for Influenza A virus nucleoprotein antigen; **D**, **H**, **L**, **P**, **T** Immunohistochemistry staining of lung sections for Influenza A virus nucleoprotein.

**Fig. 4:**
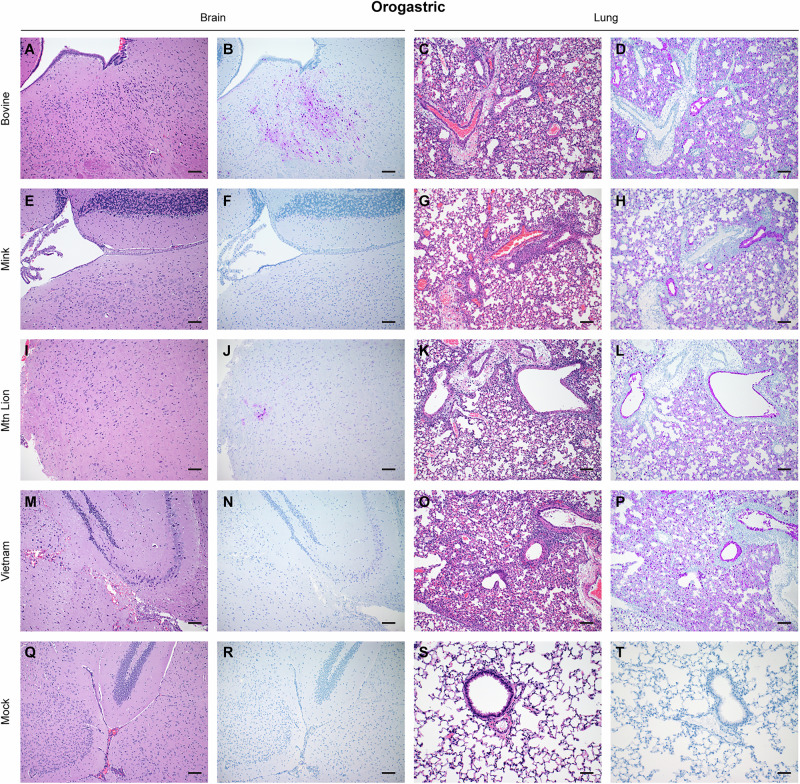
Histologic lesions and antigen expression following HPAI A(H5N1) orogastric inoculation. Six-week-old C57BL/6J mice were orogastric inoculated with 10^5^ TCID_50_ of A/bovine/OH/B24OSU-342/2024, A/mountain lion/MT/1/2024), A/mink/Spain/3691-8_22VIR10586-10/2022, or A/Vietnam/1203/2004. A subset of animals from each group (*n* = 4) was euthanized at day 4 post infection for histopathologic evaluation. Representative sections from each group shown. **A**, **E**, **I**, **M**, **Q** Hematoxylin & eosin stain of brain sections. **C**, **G**, **K**, **O**, **S** Hematoxylin & eosin stain of lung sections. **B**, **F**, **J**, **N**, **R** Immunohistochemistry staining of brain sections for Influenza A virus nucleoprotein antigen. **D**, **H**, **L**, **P**, **T** Immunohistochemistry staining of lung sections for Influenza A virus nucleoprotein.

To determine specific differences in pathogenesis by the HPAI A(H5N1) isolates in C57BL/6 J mice, we next assessed histologic lesions and anti-influenza viral nucleoprotein immunoreactivity in brain and lung samples collected on day 4 PI (Figs. 3A–T, 4A–T, Supplementary Data 1–2, Supplementary Fig. 1). Regardless of route of inoculation or isolate, no significant histopathologic changes were observed in evaluated brain sections (Figs. 3A, E, I, M, Q and 4A, E, I, M, Q). Abundant viral antigen was observed in neurons, glial cells and occasional ependymal cells (Supplementary Fig. 1A, B, F). Despite the presence of viral antigen in brain, there was no observable tissue inflammation (Figs. 3B and 4B). Interestingly, minimal virus antigen was observed in the brain of two animals inoculated with the mountain lion isolate (one of four each intranasal and orogastric) the only other isolate that caused neurologic disease in mice (Figs. 3J and 4J). No virus antigen was observed in brain tissue of mock, mink and VN1203 inoculated mice (Figs. 3F, N, R and 4F, N, R).

Bronchiolar lesions were observed in lungs from mice inoculated with the bovine, mountain lion and VN1203 isolates (Figs. 3C, K, O; 4C, K, O). In contrast, an inconsistent histopathologic picture was observed with the mink isolate. This histologic picture suggests limited pathology on day 4 PI matching  the slower disease progression observed in mice inoculated with the mink isolate.

Immunohistochemical analysis closely mirrored histopathologic observations in the lung. Virus antigen was detected in all segments of the conducting airways of all mice inoculated with the bovine and mountain lion isolates (Figs. 3D, L and 4D, L) in two of the four mice intranasally and one out of four mice orogastric inoculated with the mink isolate (Figs. 3H and 4H), in all mice intranasally inoculated with the VN1203 isolate (Fig. 3P) whereas in orogastric inoculated mice virus antigen was identified in two of the four evaluated mice (Fig. 4P). Virus antigen was detected in bronchiolar epithelial cells, type I and type II pneumocytes and alveolar macrophages of HPAI A(H5N1) inoculated animals (Supplementary Fig. 1C, D, E). As expected, virus antigen was not detected in mock infected animals (Figs. 3T and 4T). Overall, there was a strong correlation between histopathologic changes and virus antigen detection in the lung supporting high viral lung loads and respiratory disease in infected mice.

### Bovine isolate induced pro-inflammatory cytokine profile in brain tissue

**Fig. 5:**
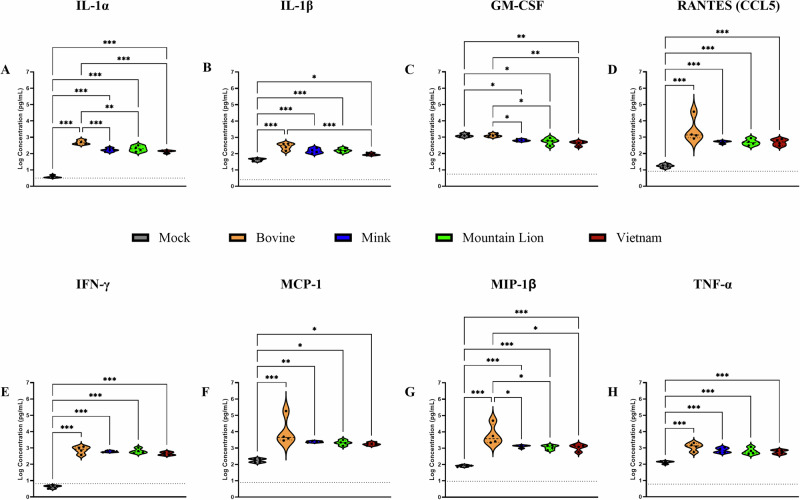
Brain cytokine expression following HPAI A(H5N1) infection. Six-week-old C57BL/6J mice (*n* = 10 per group) were inoculated with 10^5^ TCID_50_ of A/bovine/OH/B24OSU-342/2024, A/mountain lion/MT/1/2024), A/mink/Spain/3691-8_22VIR10586-10/2022, or A/Vietnam/1203/2004. A subset of animals from each group (*n* = 4) was euthanized at day 4 post infection for cytokine analysis. (**A**) Interleukin (IL)-1α, (**B**) IL-β, (**C**) granulocyte-macrophage colony-stimulating factor (GM-CSF), (**D**) regulated upon activation, normal T cell expressed and secreted (RANTES), (**E**) Interferon gamma (IFN-γ), (**F**) monocyte chemotactic protein 1 (MCP-1), (**G**) macrophage inflammatory protein-1 beta (MIP-1β), (**H**) tumor necrosis factor alpha (TNF-α). Dashed line indicates limit of detection. Statistical analyses were performed using one-way ANOVA with Tukey’s multiple comparison. ns *p* > 0.05, *p < 0.05, **p < 0.01, ****p < 0.0001. Comparisons with *p* values > 0.05 were not displayed. Violin plots show interquartile range, median and density curve.

Pathogenesis following inoculation with HPAI A(H5N1) strains has been associated with increased production of cytokines, chemokines and IFNs in humans^29^. We next determined whether the observed differences in disease course by the different HPAI A(H5N1) isolates correlated with cytokine dysregulation in our model. Since C57BL/6J mice are known to mount a predominantly Th1 response^30^, we evaluated the cytokine levels in lungs and brains of inoculated mice with a focus on the Th1 response. All HPAI A(H5N1) inoculated animals showed elevated levels of inflammatory cytokines including interleukin (IL)-1α, IL-1β, granulocyte-macrophage colony-stimulating factor (GM-CSF), regulated upon activation, normal T cell expressed and secreted (RANTES,CCL5), Interferon gamma (IFN-γ), monocyte chemotactic protein 1 (MCP-1), macrophage inflammatory protein-1 beta (MIP-1β) and tumor necrosis factor alpha (TNF-α) or Eotaxin compared to mock inoculated animals (Fig. 5, Supplementary Figs. 2–4). Consistent with the detection of infectious virus and viral antigen in the brains of C57BL/6J mice infected with the bovine isolate, significantly higher pro-inflammatory IL-1α, GM-CSF and MIP-1β cytokine levels were detected (Fig. 5).

### Bovine isolate caused more rapid disease in BALB/c mice

To verify our initial findings in C57BL/6J mice we utilized the same experimental set up with groups of BALB/c mice (*n* = 10/group). BALB/c mice were intranasally or orogastric inoculated with 10^5^ TCID_50_ of A/bovine/OH/B24OSU-342/2024, A/mountain lion/MT/1/2024, A/mink/Spain/3691-8_22VIR10586-10/2022/ and A/Vietnam/1203/04, weighed daily and monitored for signs of illness. All inoculated mice developed weight loss (Supplementary Fig. 5A, B), ruffled fur, and hunched posture followed by rapid breathing but no obvious neurologic signs. BALB/c mice inoculated with the bovine strain all succumbed a day earlier compared to inoculated C57BL/6J mice. All mice inoculated intranasally with the different isolates succumbed to infection except for a single mouse in the mink group (Supplementary Fig. 5C). Like C57BL/6J mice, orogastric inoculations with the mink, mountain lion and VN1203 isolates did not result in uniform lethality (Supplementary Fig. 5D). For comparison of viral load and cytokine dysregulation, between the two mouse strains, BALB/c mice were euthanized on day 2 PI when they had reached a similar disease stage as the inoculated C57BL/6 mice (Supplementary Figs. 6–10). In contrast to C57BL/6J mice, virus titers in brain tissue from BALB/c mice were low (Supplementary Fig. 6A, E) but all virus isolates replicated at similar levels with high titers in the lungs (Supplementary Fig. 6B, F) regardless of inoculation route. Although all the four H5N1 strains caused viremia, infectious virus was consistently detected in only the bovine-infected mice (3/4 intranasally and 4/4 orogastric animals) (Supplementary Fig. 6D, H). The H5N1 viruses induced significantly higher levels of IL-1α, IL-1β, GM-CSF, RANTES, IFN-γ, MCP-1, MIP-1α, Eotaxin compared to mock-infected BALB/c mice in lung and brain (Supplementary Figs. 7–10). Overall, all HPAI A(H5N1) isolates used here induced a significant pro-inflammatory cytokine response but minimal neuroinvasion. Virus replication revealed similar organ titers for all isolates with the highest titers in lungs. Similar to C57BL/6 J mice, disease progression in BABL/c mice infected with the bovine isolate was more advanced than with the mink, mountain lion and VN1203 isolates.

#### Low-dose inoculation with the bovine isolate confirms neurologic disease in C57BL/6J mice

**Fig. 6:**
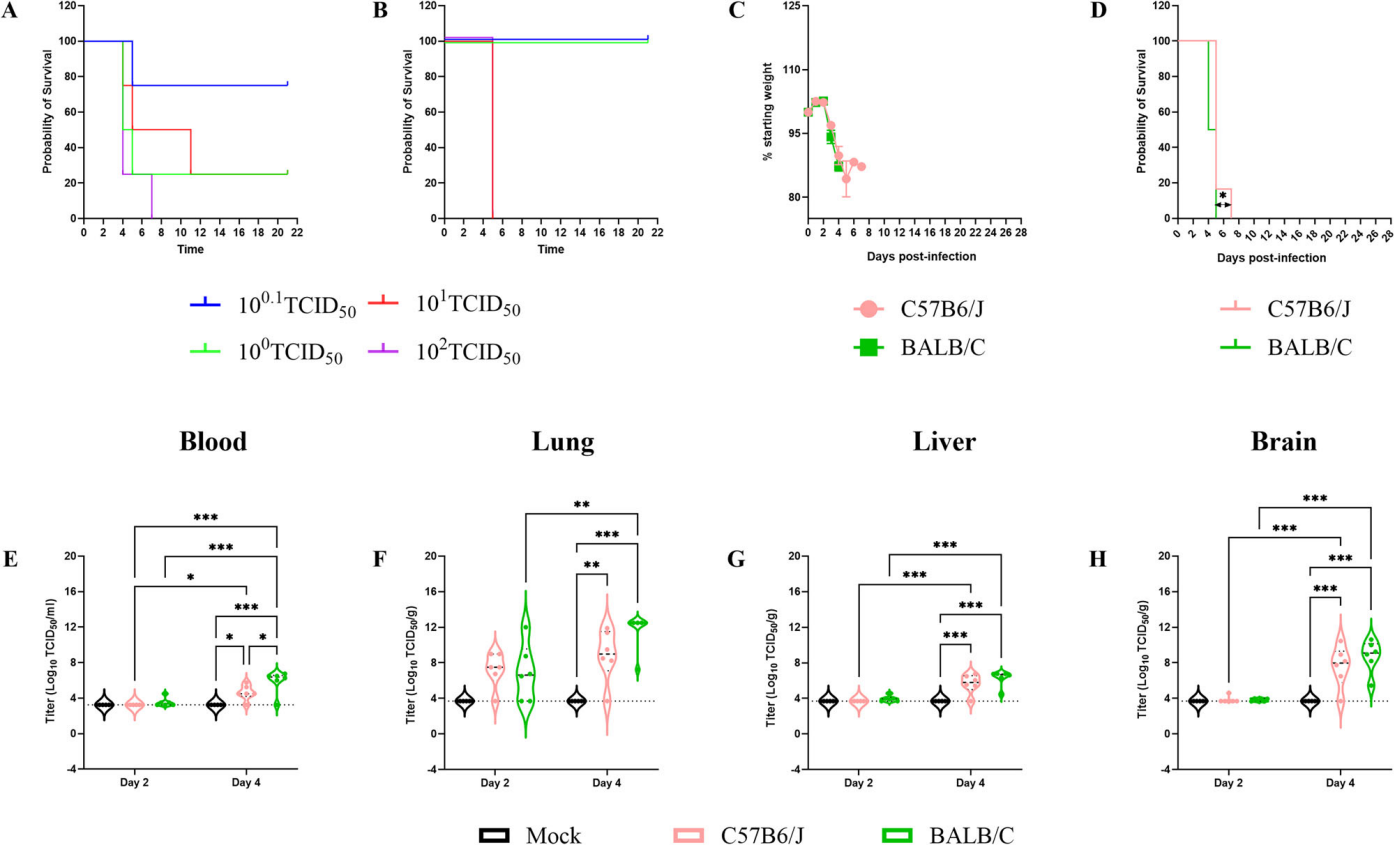
Disease progression and infectious virus titers in organs following HPAI (H5N1) infection. To determine median lethal dose (LD_50_), groups of 6-week-old female BALB/c or C57BL/6J mice were anaesthetized and intranasally inoculated with 10^0.1^, 10^0^, 10^1^, 10^2^ TCID_50_ of A/bovine/OH/B24OSU-342/2024 (H5N1) in 50 µl of DMEM without supplements. Four mice per inoculation group were monitored for survival (**A**, **B**) until day 21 post inoculation or their death, whichever was earlier. To determine viral replication kinetics and tissue tropism, groups of 18 C57BL/6 J or BALB/c mice were anaesthetized and inoculated with DMEM (mock) or 10^3^ TCID_50_ of A/bovine/OH/B24OSU-342/2024 intranasally in volume of 50 µl. Six mice per inoculation group were monitored for weight loss (**C**) survival (**D**) until day 28 post inoculation or their death, whichever was earlier. Six mice per inoculation group were euthanized at 2- or 4 days post inoculation at which blood and tissues (brain, lung, liver) were collected for virus titration using a TCID_50_ assay (**E**–**H**). Dashed line indicates limit of detection. Statistical comparisons were calculated using log-rank test with Bonferroni correction for multiple comparisons (**D**) two-way ANOVA with Tukey’s multiple comparison test (**E**–**H**) and results are indicated as *p < 0.05, **p < 0.01, ***p < 0.001, ****p < 0.0001. Comparisons with *p* values > 0.05 were not displayed. **C** Data shown as mean plus standard deviation. **E**–**H** Violin plots show interquartile range, median and density curve.

To further define virus replication and disease progression of the bovine isolate, we first determined the median mouse lethal dose (LD_50_) for the bovine isolate in both BALB/c and C57/B/6J mice via the intranasal route of inoculation. Both strains of mice were inoculated with tenfold serial dilutions of virus and monitored daily for survival (Fig. 6A, B, Supplementary Fig. 11) up to day 21. All mice inoculated with ≥10^2^ TCID_50_ of virus succumbed to infection, whereas there were varying numbers of survivors in the remaining inoculation doses resulting in LD_50_ values of 1.8 TCID_50_ in BALB/c and 3.2 TCID_50_ in C57BL/6J mice. These LD_50_ are comparable to that of VN1203 (LD_50_ 2.2 PFU)^31^ but lower than that of a related bovine isolate (A/dairy cattle/New Mexico/A240920343-93/2024, LD_50_ 31.6 PFU)^32^.

A dose of approximately 1000 LD_50_ was chosen to assess disease kinetics as it is commonly used for countermeasure efficacy studies. We inoculated BALB/c and C57BL/6J mice via the intranasal route with 10^3^ TCID_50_ of the bovine isolate. Inoculated BALB/c and C57BL/6J mice lost weight beginning day 2 post inoculation (Fig. 6C) and succumbed with meantime to death of 4.5 and 5.3 days, respectively (Fig. 6D). Survival curves between the two mouse strains were significantly different (Log-rank test *p* = 0.04). In harmony with the 10^5^ TCID_50_ study, only C57BL/6J mice displayed neurologic disease signs including tremors, circling, ataxia and hyperactivity (6/6 mice).

A subset of animals was euthanized at day 2 and 4 PI and collected blood and tissues (brain, lung, liver) to compare tissue tropism between the two mouse strains. As expected, viral titers were higher at day 4 PI (severe disease) compared to day 2 PI (disease onset) (Fig. 6E–H) in both mouse strains, but titers trended higher in BALB/c mice compared to C57BL/6 J mice at both time points. At day 2 PI replicating virus was detected mostly in lung tissue (Fig. 6F) and by day 4 PI virus was detected in blood, lung, liver and brain tissue (Fig. 6E–H) of challenged animals suggesting systematic spread of the bovine isolate. Overall, these results indicate that BALB/c mice are more susceptible to the bovine isolate than C57BL/6J mice.

**Fig. 7:**
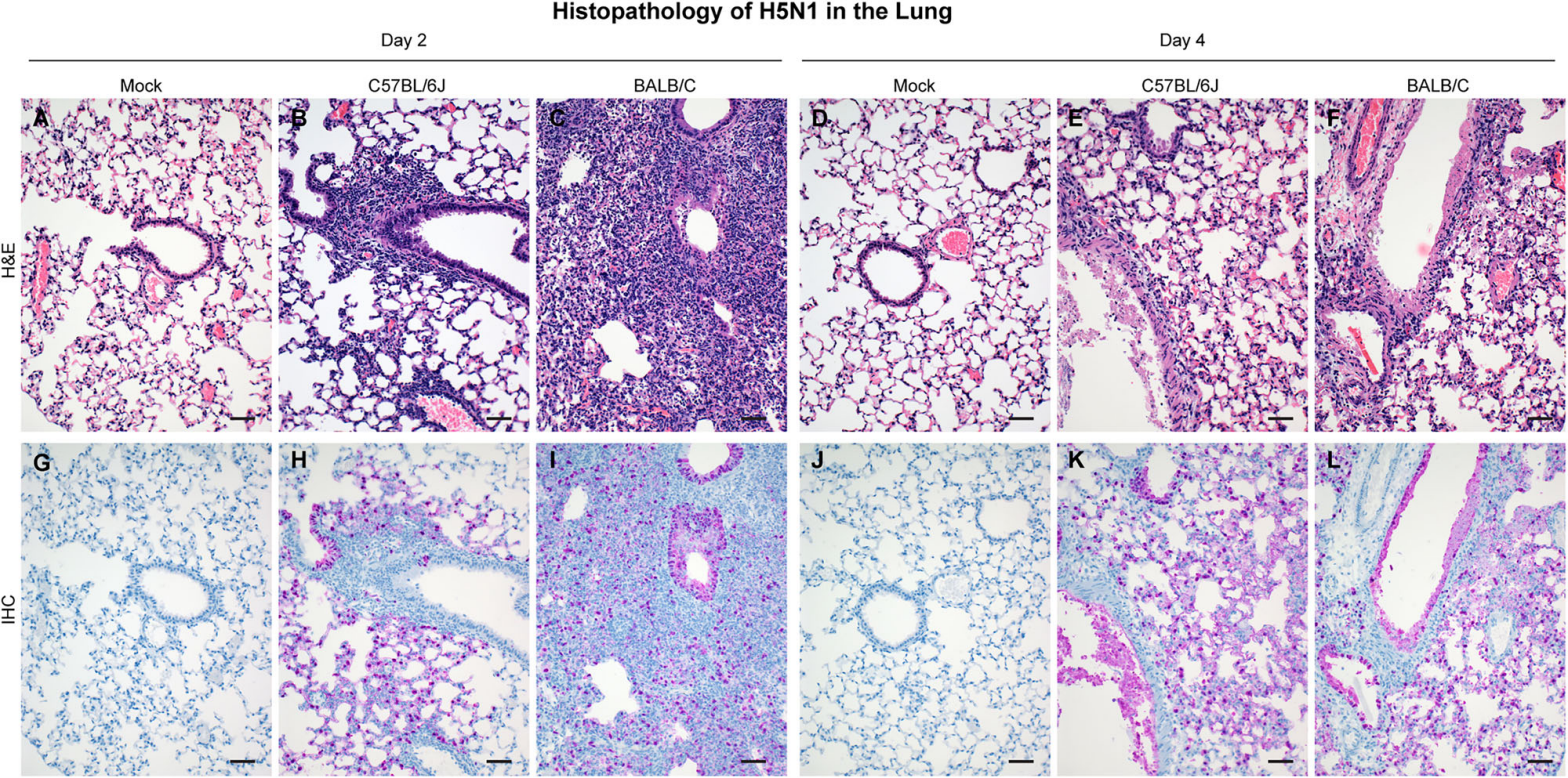
Lung histologic lesions and antigen expression following HPAI A(H5N1) inoculation. Six-week-old C57BL/6J or BALB/C mice were inoculated intranasally with DMEM (mock), or 10^3^ TCID_50_ of A/bovine/OH/B24OSU-342/2024. A subset of animals from each group (*n* = 4) was euthanized at day 2 or 4 post infection for lung histopathologic evaluation. Representative sections from each group shown. **A**–**F** Hematoxylin & eosin stain. **G**–**L** Immunohistochemistry staining for Influenza A virus nucleoprotein antigen. Magnification ×100, bar 100 µm.

**Fig. 8:**
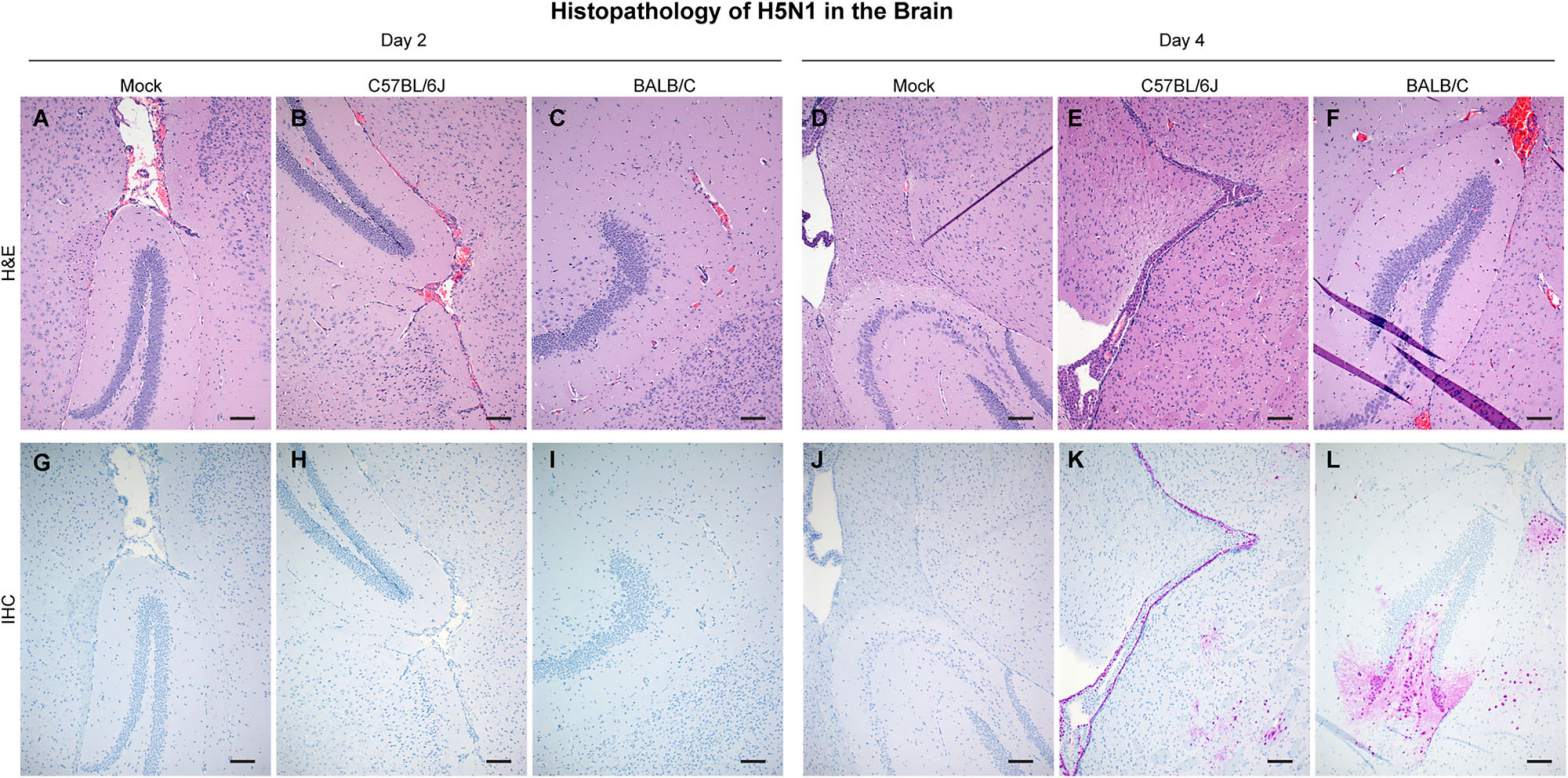
Brain histologic lesions and antigen expression following HPAI A(H5N1) inoculation. Six-week-old C57BL/6J or BALB/C mice were inoculated intranasally with DMEM (mock) or 10^3^ TCID_50_ of A/bovine/OH/B24OSU-342/2024. A subset of animals from each group (*n* = 4) was euthanized at day 2 or 4 post infection for brain histopathologic evaluation. Representative sections from each group shown. **A**–**F** Hematoxylin & eosin stain. **G**–**L** Immunohistochemistry staining for Influenza A virus nucleoprotein antigen. Magnification ×100, bar 100 µm.

Next, we assessed histologic lesions in lung and brain tissue collected on day 2 and 4 PI (Figs. 7 and 8, Supplementary Data 3). Mild to moderate necrotizing bronchiolitis with interstitial pneumonia had developed by day 2 PI in 5/6 mice in both strains (Fig. 7B, C). Consistent with the observed pathology, viral antigen was detected by day 2 PI in these animals (Fig. 7H, I). Tissue damage was more severe by day 4 PI; segmental bronchiolitis and denuding of the entire bronchiolar mucosa in 5/6 mice in both BALB/c and C57BL/6J mice was observed. Mild to moderate interstitial pneumonia with severe vasculitis was also notable (Fig. 7E, F) and viral antigen was detected in 5/6 C57BL/6 J mice and 6/6 BALB/c mice at day 4 PI (Fig. 7K, L). Analysis of the brain tissue revealed no histologic lesion or viral antigen at day 2 PI in either mouse strains (Fig. 8B, C, H, I). Similar to the 10^5^ TCID_50_ dose study, abundant viral antigen staining in neurons and glial cells was observed in both mouse strains despite no marked histopathological change at day 4 PI (Fig. 8E, F, K, L).

## Discussion

Recent increases in cases of HPAI A(H5N1) clade 2.3.4.4b virus infections in mammals including humans is concerning for animal and public health^33^. Human cases in the United States remain mild and limited in number but continued circulation with incidental spillover into mammalian populations^8–10^ could confer mutations resulting in more efficient transmission and increased virulence in mammals including humans.

Our mouse studies have demonstrated increased virulence of a recent bovine isolate relative to  the reference VN1203 and two distinct recent HPAI A(H5N1) isolates (mink and mountain lion) in two mouse strains. The bovine isolate caused systemic infection with increased capacity for neuroinvasion and neurovirulence a trait that is not unfamiliar with HPAI A(H5N1) clade 2.3.4.4b viruses^4,27,34^. In addition, differences in neurotropism for H5N1 strains have already been previously reported^35^. The disease course observed in C57BL/6J mice inoculated with the bovine isolate is a consequence of combined neurological and respiratory disease. High viral load, antigen staining and an elevated pro-inflammatory cytokine profile in brain tissue further support neurological disease manifestation associated with the bovine isolate. There was some evidence of neurological involvement with the mountain lion isolate as two mice displayed both neurologic disease signs and antigen staining in the brain. Noteworthy, the mountain lion and bovine isolates are genetically more closely related.

The mechanism of neuroinvasion of the bovine isolate remains undetermined but the presence of viremia may favor hematogenous spread via the blood-brain-barrier. Indeed, bloodborne spread of an H5N1 isolate and a mouse-adapted variant of H7N7 has been documented^36,37^. Neuroinvasion through cranial nerves, which seems plausible for the intranasal route of infection, also remains a viable option, and both neuroinvasion through the blood-brain-barrier and cranial nerves have been demonstrated in ferrets infected with H5N1^38^. Further investigations are needed to understand virus replication and spread for the orogastric route of infection, although submucosal and myenteric plexi in the intestine could serve as sites of virus entry. We cannot, however, exclude that the mice may have been unintendedly co-exposed via the respiratory tract during orogastric inoculation.

Despite achieving comparable viral burden in brain tissues, C57BL/6J mice were more prone to developing neurologic disease signs compared to BALB/c mice when inoculated with low a dose of the bovine isolate. Neurologic disease in C57BL/6J mice infected with the bovine isolate has been reported confirming our results here^39^. Furthermore, neurotropism of a bovine HPAI A(H5N1) clade2.3.4.4b isolate in BALB/c mice was recently reported without mentioning of neurologic disease signs^32^. Our observations here  could be a consequence of a slightly advanced virus replication in BALB/c over C57BL/6J mice leading to fatal respiratory disease before neurologic disease develops. Conversely, there may be a distinct organ tropism and disease progression associated with the bovine isolate in the two mouse strains. BALB/c and C57BL/6J mice differ in their immune responses with C57BL/6J mice demonstrating a biased Th1 and M-1 response and BALB/c mice showing a dominant Th2 and M-2 response^30,40^. These differences in immune responses have been discussed for differences in susceptibility of these mice to the 2009 pandemic H1N1 influenza virus and a human H5N1 isolate^41^. Currently, it remains unclear whether differences in baseline immune responses influence disease progression and pathology of current H5N1 isolates.

High viral load and viral antigen staining in brain tissue indicated replication of the bovine isolate in the central nervous system. The absence of histological lesions, however, was intriguing as infected mice developed neurological signs. Given the lack of detectable inflammatory infiltrates and similar lack of neuronal necrosis, neuronal metabolic disturbances are deemed the most probable explanation for the described clinical signs. Even in the absence of gross brain lesions, subtle brain damage is a possibility and may require further analysis. The proinflammatory phenotype found in brain tissue may support this notion. Necrosis and inflammation in brain tissue notably in the cerebrum has been reported before in H5N1 infected mammals^4,36^ which we did not observe here.

Certain molecular markers/motifs have been reported to influence the replicative capacity, virulence, and transmission of influenza A viruses in both poultry and mammals^42,43^. All recent HPAI H5N1 isolates used here still possess the full-length neuraminidase (NA) stalk region, which is lacking in the VN1203 isolate. Deletion of this stalk region has been associated with increased virulence in avian species and mice^44–47^. Similarly, a 5-amino acid deletion in the nonstructural protein NS1 linker region connecting the RNA binding domain with the effector domain has been reported to confer increased viral replication potential in chickens and mice^48–50^. Again, this deletion is not present in the three recent isolates in contrast to VN1203 indicating that other mutations may be equally or more important for virulence in mice. Furthermore, the E627K substitution in PB2, one of the polymerase complex proteins, of avian-origin influenza viruses is thought to support more efficient replication in mammalian species including mice^51,52^. In contrast to VN1203, none of the recent HPAI A(H5N1) isolates used in this study possesses this substitution despite similar or higher virulence in mice. On the other hand, all HPAI A(H5N1) isolates evaluated in the study harbor mutations in the matrix protein M1 (such as 30D, 43M, and 215A)^42,53,54^ and NS1 (such as 42S, 103F, and 106M)^55^ known to increase virulence in mice.

A previous study did not report increased pathogenicity of the bovine isolate compared to the VN1203 isolate in BALB/c mice^32^ contrary to our findings here. The different experimental outcomes may be explained by the experimental variations between the two studies, including the study design and the virus isolates under study. We used A/bovine/OH/B24OSU-342/2024 in our experiments, Eisfeld et al.^32^ utilized the A/dairy cattle/New Mexico/A240920343-93/2024 (New Mexico). While these two isolates are in the same clade, they may well exhibit distinct characteristics in disease manifestations. It would be interesting to compare the newly emerged isolates directly under similar conditions.

In these studies, we found the bovine isolate to spread more readily to other tissues after orogastric and intranasal infection while systemic infection was not obvious with the mink, mountain lion and VN1203 isolates showing only insignificant and inconsistent viremia in individual infected animals. It is also unclear why lethality after orogastric inoculation was observed only with the bovine isolate. Interestingly, amino acid comparison shows that the mink, mountain lion, New Mexico and VN1203 isolates harbor the 497K in the polymerase acidic (PA) protein. Mutant H7N9 viruses possessing the 497K displayed significantly lower lung viral titers and increased LD_50_ in mice compared to wild-type viruses^56^ suggesting that the amino acid change decreased polymerase activity and virulence. Future studies need to address the role of sequence differences to understand and define molecular signatures that explain the increased virulence and altered organ tropism of the bovine isolate in mammalian species.

Our study defined orogastric inoculations as a potent exposure route for HPAI A(H5N1) clade 2.3.4.4b viruses. This offers a plausible explanation for reported infections in wild and domesticated carnivores with HPAI A(H5N1) viruses that may get infected through hunting and scavenging as may be the case for the mountain lion from which one of the viruses used here was isolated. In addition, orogastric exposure may be a viable infection route for other mammals, such as cats and humans, when consuming raw milk from infected dairy cows. Orogastric exposure resulted in subclinical infection in monkeys^57^ and could be a route supporting cattle-to-cattle transmission.

In conclusion, the bovine isolate showed high virulence for mice when inoculated by the intranasal and orogastric routes. C57BL/6J and BALB/c mice provide excellent in vivo screening models for efficacy testing of antiviral, therapeutic and vaccine candidates against infections with these emerging HPAI A(H5N1) viruses. The C57BL/6J model allows studies on mechanisms of neuroinvasion and neurovirulence of the emerging HPAI A(H5N1) viruses. Both mouse models may be used to gain deeper insight into infection by the orogastric route, an exposure route of importance for cow-to-human as well as bird/poultry-to-carnivore transmission of emerging HPAI A(H5N1) isolates. Thus, the mouse models will be extremely beneficial for animal and public health responses to the current HPAI A(H5N1) outbreak.

## Methods

### Biosafety and ethics

All infectious work was conducted in a maximum containment laboratory  following operating procedures approved by the Institutional Biosafety Committee of the Rocky Mountain Laboratories, Division of Intramural Research, National Institute of Allergy and Infectious Diseases, National Institutes of Health (Hamilton, MT, USA). Animal experiments were approved by the Rocky Mountain Laboratories Animal Care and Use Committee (ACUC) (protocol number 2024-19) and carried out in an AALAC accredited facility. Six-week-old C57BL/6J and BALB/c mice were purchased from Jackson laboratories and randomly assigned to study groups. Animals were housed under controlled conditions of 30–35% humidity, 22 °C temperature and light (12-h light/12-h dark cycles). Food and water were provided ad libitum. All procedures on mice were performed under anesthesia. Sample inactivation followed established protocols approved by the RML Institutional Biosafety Committee.

### Viruses and cells

The highly pathogenic H5N1 virus strains used in this study include A/Vietnam/1203/2004, A/bovine/OH/B24OSU-342/2024, A/mink/Spain/3691-8_22VIR10586-10/2022^16^ and A/mountain lion/MT/1/2024^23^. Virus growth and titration were performed on Madin-Darby canine kidney (MDCK) cells in Minimal Essential Media (MEM) (Sigma-Aldrich, St. Louis, MO) containing 4 µg/ml trypsin, 2 mM l-glutamine, 50 U/mL penicillin, 50 µg/ml streptomycin, 1% NEAA, 20 mM HEPES (all Thermo Fisher Scientific, Waltham, MA). MDCK cells were maintained in MEM supplemented with 10% fetal bovine serum (FBS) (Wisent Inc., St. Bruno, Canada), 2 mM l-glutamine, 50 U/mL penicillin, 50μg/ml streptomycin, 1% non-essential amino acids (NEAA) and 20 mM HEPES.

#### 10^5^ TCID_50_ dose HPAI A(H5N1) pathogenesis study

Ten mice per virus group were anaesthetized with isoflurane and inoculated with DMEM (mock), or DMEM containing 10^5^ TCID_50_ of A/bovine/OH/B24OSU-342/2024, A/mink/Spain/3691-8_22VIR10586-10/2022, A/mountain lion/MT/1/2024) and A/Vietnam/1203/04 intranasally or orogastric in volumes of 50 µl and 250 µl, respectively. For orogastric inoculation, inoculum was deposited into the stomach using a feeding tube. All animals were weighed daily and monitored for clinical signs of disease. Hyperactivity was defined as excessive movement upon observation. In this case, mice would circle the cage repeatedly without stimulation or rest. Four animals per inoculation group were euthanized on day 2 post infection (BALB/c mice) or day 4 post infection (C57BL/6J mice) at which time blood and tissues (brain, lung, liver) were collected for virus titration and histopathology. Six mice per inoculation group were monitored for survival until day 28 post inoculation, anesthetized, bled and humanely euthanized. No sex-specific differences in disease outcome were observed.

#### Determination of the median lethal dose (LD_50_)

To determine the LD_50_, groups of four 6-week-old female BALB/c or C57BL/6J mice were anaesthetized by inhalational isoflurane and intranasally inoculated with 10^0.1^, 10^0^, 10^1^, 10^2^ TCID_50_ in 50 µl DMEM respectively of A/bovine/OH/B24OSU-342/2024 (H5N1). Animals were monitored daily for disease progression, body weight changes and survival for 21 days. The LD_50_ values were calculated according to the method of Reed and Muench^58^.

#### 10^3^ TCID_50_ HPAI A(H5N1) pathogenesis study

Groups of 18 six-week-old C57BL/6J or BALB/c mice were anaesthetized by inhalational isoflurane and intranasally inoculated with DMEM (mock) or DMEM containing 10^3^ TCID_50_ of A/bovine/OH/B24OSU-342/2024 in 50 µl volume. All animals were monitored daily for clinical signs of disease and weight loss. Six animals per inoculation group were euthanized on day 2 or day 4 PI at which time blood and tissues (brain, lung, liver) were collected for virus titration and histopathology. Six mice per inoculation group were monitored for disease progression, body weight changes and survival outcomes until day 28 post inoculation anesthetized, bled and humanely euthanized.

### Virus titration

Infectious virus was quantified on MDCK cells. MDCK cells were seeded in 96-well plates in MEM supplemented with 10% FBS, 2 mM l-glutamine, 50 U/mL penicillin, 50 μg/ml streptomycin, 1% NEAA and 20 mM HEPES. Mouse tissue specimens in tubes containing steel beads and 1 mL infection media (MEM containing 4 μg/ml trypsin, 2 mM l-glutamine, 50 U/mL penicillin, 50 μg/ml streptomycin, 1× NEAA and 20 mM HEPES) were homogenized using the TissueLyser II (Qiagen). Blood samples were initially diluted (1:10) in phosphate-buffered saline without Ca2^+^/Mg2^+^. Subsequent ten-fold serial dilutions were performed in media. A 100 μL aliquot of diluted sample was applied on MDCK cells and incubated for 3 days at 37 °C. Subsequently, 75 µl of 0.33% Turkey erythrocytes were added to 25 µl aliquot of the supernatant from infected MDCK cells in 96-well plates and incubated for 1 h at 37 °C before hemagglutination was read. TCID_50_ was calculated using the Reed & Muench method^58^.

### Cytokine analysis

Brain and lung tissue samples were thawed and homogenized in 1 ml of phosphate-buffered saline using a TissueLyser II at 30-Hz oscillation frequency for 5 min. Homogenates were centrifuged (10,000 × *g* for 5 min) to remove debris and irradiated according to approved procedures to inactivate HPAI H5N1 virus^59^. Cleared supernatants were used for cytokine quantification using the Bio-Plex Pro^TM^ Mouse Cytokine 23-plex Assay (Biorad) according to the manufacturer’s instructions.

### Histopathology and Immunohistochemistry

Tissues were fixed in 10% Neutral Buffered Formalin x2 changes, for a minimum of 7 days. Tissues were placed in cassettes and processed with a Sakura VIP-6 Tissue Tek, on a 12-h automated schedule, using a graded series of ethanol, xylene, and ParaPlast Extra. Embedded tissues were sectioned at 5 um and dried overnight at 42 degrees Celsius prior to staining. Immunoreactivity was detected using Millipore Sigma Anti-Influenza A nucleoprotein antibody (Cat. #ABF1820-25UL) at a 1:12,000 dilution. Roche Tissue Diagnostics DISCOVERY Omnimap anti-rabbit HRP (#760-4311) was used as a secondary antibody. For negative controls, replicate sections from each control block were stained in parallel following an identical protocol, with the primary antibody replaced by Vector Laboratories rabbit IgG (#I-1000-5) at a 1:2500 dilution. The tissues were stained using the DISCOVERY ULTRA automated stainer (Ventana Medical Systems) with a Roche Tissue Diagnostics DISCOVERY purple kit (#760-229). One slide was examined per animal per tissue. Histologic lesions were scored by a board-certified pathologist blinded to the animal study groups.

### Amino acid sequence analysis

Amino acid identity of all the proteins of HPAI H5N1 isolate VN1203, Mink, Mountain Lion, and Bovine were analyzed using Clustal Omega, a multiple sequence alignment tool^60^.

### Statistical analysis

Statistical analysis was performed using GraphPad Prism version 10.3.0 for Windows (GraphPad Software, San Diego, CA, USA, www.graphpad.com, 6 May 2025 ). Differences between mouse groups were compared using one-way ANOVA with Turkey’s multiple comparison. Survival curves were compared by using the log-rank Mantel–Cox test. Statistical significance was set at *p* < 0.05.

### Reporting summary

Further information on research design is available in the Nature Portfolio Reporting Summary linked to this article.

## Supplementary information

Supplementary Information

Description of Additional Supplementary Files

Supplementary Data 1

Supplementary Data 2

Supplementary Data 3

Reporting Summary

Transparent Peer Review file

## Source data

Source data

## Data Availability

All relevant data are within the manuscript and its Supporting Information files. Source data are provided with this paper.

## References

[1] Chen, H. et al. Establishment of multiple sublineages of H5N1 influenza virus in Asia: implications for pandemic control. *Proc. Natl Acad. Sci. USA* **103**, 2845–2850 (2006). 16473931 10.1073/pnas.0511120103 PMC1413830

[2] WHO/OIE/FAO H5N1 Evolution Working Group. Continued evolution of highly pathogenic avian influenza A (H5N1): updated nomenclature. *Influenza Other Respir. Viruses* **6**, 1–5 (2012). 22035148 10.1111/j.1750-2659.2011.00298.x PMC5074649

[3] Xie, R. P. et al. The episodic resurgence of highly pathogenic avian influenza H5 virus. *Nature* **622**, 810–817 (2023). 37853121 10.1038/s41586-023-06631-2

[4] Elsmo, E. J. et al. Highly pathogenic avian influenza A(H5N1) virus clade 2.3.4.4b infections in wild terrestrial mammals, United States, 2022. *Emerg. Infect. Dis.* **29**, 2451–2460 (2023). 37987580 10.3201/eid2912.230464 PMC10683806

[5] Burrough, E. R. et al. Highly pathogenic avian influenza A(H5N1) clade 2.3.4.4b virus infection in domestic dairy cattle and cats, United States, 2024. *Emerg. Infect. Dis.* **30**, 1335–1343 (2024). 38683888 10.3201/eid3007.240508 PMC11210653

[6] H5 bird flu: current situation*.* https://www.cdc.gov/bird-flu/situation-summary/index.html (2025).

[7] CDC. CDC A(H5N1) bird flu response update August 2, 2024, https://www.cdc.gov/bird-flu/spotlights/h5n1-response-08022024.html (2024).

[8] Garg, S. et al. Outbreak of highly pathogenic avian influenza A(H5N1) viruses in U.S. dairy cattle and detection of two human cases - United States, 2024. *MMWR Morb. Mortal. Wkly Rep.* **73**, 501–505 (2024). 38814843 10.15585/mmwr.mm7321e1 PMC11152367

[9] Morse, J. et al. Influenza A(H5N1) virus infection in two dairy farm workers in Michigan. *N. Engl. J. Med.* **391**, 963–964 (2024). 39115081 10.1056/NEJMc2407264

[10] Uyeki, T. M. et al. Highly pathogenic avian influenza A(H5N1) virus infection in a dairy farm worker. *N. Engl. J. Med* **390**, 2028–2029 (2024). 38700506 10.1056/NEJMc2405371

[11] Garg, S. et al. Highly pathogenic avian influenza A(H5N1) virus infections in humans. *N. Engl. J. Med.*10.1056/NEJMoa2414610 (2024). 10.1056/NEJMoa2414610 39740051

[12] Signs and symptoms of bird flu in people. https://www.cdc.gov/bird-flu/signs-symptoms/index.html (2025).

[13] CDC confirms human cases of H5 bird flu among colorado poultry workers. https://www.cdc.gov/media/releases/2024/p-0715-confirm-h5.html (2024).

[14] CDC confirms second human H5 bird flu case in Michigan; third case tied to dairy outbreak. https://www.cdc.gov/media/releases/2024/p0530-h5-human-case-michigan.html (2024).

[15] Jassem, A. N. et al. Critical illness in an adolescent with influenza A(H5N1) virus infection. *N. Engl. J. Med.*10.1056/NEJMc2415890 (2024). 10.1056/NEJMc2415890 39740022

[16] Aguero, M. et al. Highly pathogenic avian influenza A(H5N1) virus infection in farmed minks, Spain, October 2022. *Euro Surveill*. **28**. 10.2807/1560-7917.ES.2023.28.3.2300001 (2023). 10.2807/1560-7917.ES.2023.28.3.2300001 PMC9853945 36695488

[17] Kareinen, L. et al. Highly pathogenic avian influenza A(H5N1) virus infections on fur farms connected to mass mortalities of black-headed gulls, Finland, July to October 2023. *Euro Surveill.* **29**. 10.2807/1560-7917.ES.2024.29.25.2400063 (2024). 10.2807/1560-7917.ES.2024.29.25.2400063 PMC11191417 38904109

[18] Lindh, E. et al. Highly pathogenic avian influenza A(H5N1) virus infection on multiple fur farms in the South and Central Ostrobothnia regions of Finland, July 2023. *Euro Surveill.* **28**. 10.2807/1560-7917.ES.2023.28.31.2300400 (2023). 10.2807/1560-7917.ES.2023.28.31.2300400 PMC10401912 37535475

[19] Domanska-Blicharz, K. et al. Outbreak of highly pathogenic avian influenza A(H5N1) clade 2.3.4.4b virus in cats, Poland, June to July 2023. *Euro Surveill.* **28**. 10.2807/1560-7917.ES.2023.28.31.2300366 (2023). 10.2807/1560-7917.ES.2023.28.31.2300366 PMC10401911 37535474

[20] Munoz, G. et al. Stranding and mass mortality in humboldt penguins (Spheniscus humboldti), associated to HPAIV H5N1 outbreak in Chile. *Prev. Vet. Med.* **227**, 106206 (2024). 38696942 10.1016/j.prevetmed.2024.106206

[21] Uhart, M. M. et al. Epidemiological data of an influenza A/H5N1 outbreak in elephant seals in Argentina indicates mammal-to-mammal transmission. *Nat. Commun.* **15**, 9516 (2024). 39528494 10.1038/s41467-024-53766-5 PMC11555070

[22] Leguia, M. et al. Highly pathogenic avian influenza A (H5N1) in marine mammals and seabirds in Peru. *Nat. Commun.* **14**, 5489 (2023). 37679333 10.1038/s41467-023-41182-0 PMC10484921

[23] Kaiser, F. et al. Inactivation of avian influenza A(H5N1) virus in raw milk at 63 °C and 72 °C. *N. Engl. J. Med.*10.1056/NEJMc2405488 (2024). 10.1056/NEJMc2405488 PMC11463970 38875103

[24] Sillman, S. J., Drozd, M., Loy, D. & Harris, S. P. Naturally occurring highly pathogenic avian influenza virus H5N1 clade 2.3.4.4b infection in three domestic cats in North America during 2023. *J. Comp. Pathol.* **205**, 17–23 (2023). 37586267 10.1016/j.jcpa.2023.07.001

[25] Rijks, J. M. et al. Highly pathogenic avian influenza A(H5N1) virus in wild red foxes, the Netherlands, 2021. *Emerg. Infect. Dis.* **27**, 2960–2962 (2021). 34670656 10.3201/eid2711.211281 PMC8544991

[26] Puryear, W. et al. Highly pathogenic avian influenza A(H5N1) virus outbreak in New England seals, United States. *Emerg. Infect. Dis.* **29**, 786–791 (2023). 36958010 10.3201/eid2904.221538 PMC10045683

[27] Cronk, B. D. et al. Infection and tissue distribution of highly pathogenic avian influenza A type H5N1 (clade 2.3.4.4b) in red fox kits (Vulpes vulpes). *Emerg. Microbes Infect.* **12**, 2249554 (2023). 37589241 10.1080/22221751.2023.2249554 PMC10512766

[28] Kirk, N. M., Liang, Y. & Ly, H. Comparative pathology of animal models for influenza A virus infection. *Pathogens* **13**. 10.3390/pathogens13010035 (2023). 10.3390/pathogens13010035 PMC10820042 38251342

[29] Korteweg, C. & Gu, J. Pathology, molecular biology, and pathogenesis of avian influenza A (H5N1) infection in humans. *Am. J. Pathol.* **172**, 1155–1170 (2008). 18403604 10.2353/ajpath.2008.070791 PMC2329826

[30] Fornefett, J. et al. Comparative analysis of humoral immune responses and pathologies of BALB/c and C57BL/6 wildtype mice experimentally infected with a highly virulent Rodentibacter pneumotropicus (Pasteurella pneumotropica) strain. *BMC Microbiol* **18**, 45 (2018). 29848308 10.1186/s12866-018-1186-8 PMC5977748

[31] Maemura, T. et al. Characterization of highly pathogenic clade 2.3.4.4b H5N1 mink influenza viruses. *Ebiomedicine* **97**, 104827 (2023). 37812908 10.1016/j.ebiom.2023.104827 PMC10579283

[32] Eisfeld, A. J. et al. Pathogenicity and transmissibility of bovine H5N1 influenza virus. *Nature.*10.1038/s41586-024-07766-6 (2024). 10.1038/s41586-024-07766-6 PMC11390473 38977017

[33] Neumann, G. & Kawaoka, Y. Highly pathogenic H5N1 avian influenza virus outbreak in cattle: the knowns and unknowns. *Nat. Rev. Microbiol*. 10.1038/s41579-024-01087-1 (2024). 10.1038/s41579-024-01087-1 PMC12720498 39060613

[34] Bauer, L., Benavides, F. F. W., Veldhuis Kroeze, E. J. B., de Wit, E. & van Riel, D. The neuropathogenesis of highly pathogenic avian influenza H5Nx viruses in mammalian species including humans. *Trends Neurosci.* **46**, 953–970 (2023). 37684136 10.1016/j.tins.2023.08.002 PMC10591965

[35] Nishimura, H., Itamura, S., Iwasaki, T., Kurata, T. & Tashiro, M. Characterization of human influenza A (H5N1) virus infection in mice: neuro-, pneumo- and adipotropic infection. *J. Gen. Virol.* **81**, 2503–2510 (2000). 10993940 10.1099/0022-1317-81-10-2503

[36] Rimmelzwaan, G. F. et al. Influenza A virus (H5N1) infection in cats causes systemic disease with potential novel routes of virus spread within and between hosts. *Am. J. Pathol.* **168**, 176–183 (2006). 16400021 10.2353/ajpath.2006.050466 PMC1592682

[37] Gabriel, G. et al. Spread of infection and lymphocyte depletion in mice depends on polymerase of influenza virus. *Am. J. Pathol.* **175**, 1178–1186 (2009). 19700749 10.2353/ajpath.2009.090339 PMC2731136

[38] Schrauwen, E. J. A. et al. The multibasic cleavage site in H5N1 virus is critical for systemic spread along the olfactory and hematogenous routes in ferrets. *J. Virol.* **86**, 3975–3984 (2012). 22278228 10.1128/JVI.06828-11 PMC3302532

[39] Mostafa, A. et al. Replication kinetics, pathogenicity and virus-induced cellular responses of cattle-origin influenza A(H5N1) isolates from Texas, United States. *Emerg Microbes Infec.* **14**. 10.1080/22221751.2024.2447614 (2025). 10.1080/22221751.2024.2447614 PMC11721806 39727152

[40] Mills, C. D., Kincaid, K., Alt, J. M., Heilman, M. J. & Hill, A. M. M-1/M-2 macrophages and the Th1/Th2 paradigm. *J. Immunol.* **164**, 6166–6173 (2000). 28923981 10.4049/jimmunol.1701141

[41] Otte, A. et al. Differential host determinants contribute to the pathogenesis of 2009 pandemic H1N1 and human H5N1 influenza A viruses in experimental mouse models. *Am. J. Pathol.* **179**, 230–239 (2011). 21703405 10.1016/j.ajpath.2011.03.041 PMC3123800

[42] Suttie, A. et al. Inventory of molecular markers affecting biological characteristics of avian influenza A viruses. *Virus Genes* **55**, 739–768 (2019). 31428925 10.1007/s11262-019-01700-z PMC6831541

[43] Liang, Y. Pathogenicity and virulence of influenza. *Virulence* **14**, 2223057 (2023). 37339323 10.1080/21505594.2023.2223057 PMC10283447

[44] Stech, O. et al. The neuraminidase stalk deletion serves as major virulence determinant of H5N1 highly pathogenic avian influenza viruses in chicken. *Sci. Rep.* **5**. 10.1038/srep13493 (2015). 10.1038/srep13493 PMC4549673 26306544

[45] Matsuoka, Y. et al. Neuraminidase stalk length and additional glycosylation of the hemagglutinin influence the virulence of influenza H5N1 viruses for mice. *J. Virol.* **83**, 4704–4708 (2009). 19225004 10.1128/JVI.01987-08 PMC2668507

[46] Sun, Y. et al. Amino acid 316 of hemagglutinin and the neuraminidase stalk length influence virulence of H9N2 influenza virus in chickens and mice. *J. Virol.* **87**, 2963–2968 (2013). 23269805 10.1128/JVI.02688-12 PMC3571412

[47] Zhou, H. et al. The special neuraminidase stalk-motif responsible for increased virulence and pathogenesis of H5N1 influenza A virus. *PLoS ONE* **4**, e6277 (2009). 19609439 10.1371/journal.pone.0006277 PMC2707603

[48] Long, J. X., Peng, D. X., Liu, Y. L., Wu, Y. T. & Liu, X. F. Virulence of H5N1 avian influenza virus enhanced by a 15-nucleotide deletion in the viral nonstructural gene. *Virus Genes* **36**, 471–478 (2008). 18317917 10.1007/s11262-007-0187-8

[49] Trapp, S. et al. Shortening the unstructured, interdomain region of the non-structural protein NS1 of an avian H1N1 influenza virus increases its replication and pathogenicity in chickens. *J. Gen. Virol.* **95**, 1233–1243 (2014). 24694396 10.1099/vir.0.063776-0

[50] Rosario-Ferreira, N., Preto, A. J., Melo, R., Moreira, I. S. & Brito, R. M. M. The central role of non-structural protein 1 (NS1) in influenza biology and infection. *Int. J. Mol. Sci*. **21**. 10.3390/ijms21041511 (2020). 10.3390/ijms21041511 PMC7073157 32098424

[51] Subbarao, E. K., London, W. & Murphy, B. R. A single amino-acid in the Pb2-gene of influenza-A virus is a determinant of host range. *J. Virol.* **67**, 1761–1764 (1993). 8445709 10.1128/jvi.67.4.1761-1764.1993 PMC240216

[52] Hatta, M., Gao, P., Halfmann, P. & Kawaoka, Y. Molecular basis for high virulence of Hong Kong H5N1 influenza A viruses. *Science* **293**, 1840–1842 (2001). 11546875 10.1126/science.1062882

[53] Fan, S. et al. Two amino acid residues in the matrix protein M1 contribute to the virulence difference of H5N1 avian influenza viruses in mice. *Virology* **384**, 28–32 (2009). 19117585 10.1016/j.virol.2008.11.044

[54] Nao, N. et al. A single amino acid in the M1 protein responsible for the different pathogenic potentials of H5N1 Highly pathogenic avian influenza virus strains. *PLoS ONE* **10**, e0137989 (2015). 26368015 10.1371/journal.pone.0137989 PMC4569272

[55] Jiao, P. et al. A single-amino-acid substitution in the NS1 protein changes the pathogenicity of H5N1 avian influenza viruses in mice. *J. Virol.* **82**, 1146–1154 (2008). 18032512 10.1128/JVI.01698-07 PMC2224464

[56] Yamayoshi, S. et al. Enhanced replication of highly pathogenic influenza A(H7N9) virus in humans. *Emerg. Infect. Dis.* **24**, 746–750 (2018). 29553313 10.3201/eid2404.171509 PMC5875272

[57] Rosenke, K. et al. Pathogenesis of bovine H5N1 clade 2.3.4.4b infection in Macaques. *Nature.*10.1038/s41586-025-08609-8 (2025). 10.1038/s41586-025-08609-8 39814072

[58] Reed, L. J. & Muench, H. A simple method of estimating fifty percent endpoints. *Am. J. Epidemiol.* **27**, 493–497 (1938).

[59] Feldmann, F., Shupert, W. L., Haddock, E., Twardoski, B. & Feldmann, H. Gamma irradiation as an effective method for inactivation of emerging viral pathogens. *Am. J. Tropical Med. Hyg.* **100**, 1275–1277 (2019). 10.4269/ajtmh.18-0937 PMC6493948 30860018

[60] Sievers, F. et al. Fast, scalable generation of high-quality protein multiple sequence alignments using Clustal Omega. *Mol. Syst. Biol.* **7**, 539 (2011). 21988835 10.1038/msb.2011.75 PMC3261699

