# Supplementary material for: Highly pathogenic avian influenza H5N1 clade 2.3.4.4b genotype B3.13 is highly virulent for mice, rapidly causing acute pulmonary and neurologic disease
Source: Nat Commun. 2025 Jul 1;16:5738. doi: 10.1038/s41467-025-60407-y (PMC12216816; doi:10.1038/s41467-025-60407-y)
Supplement: Supplementary file 1 — Supplementary Information [file 41467_2025_60407_MOESM1_ESM.pdf]

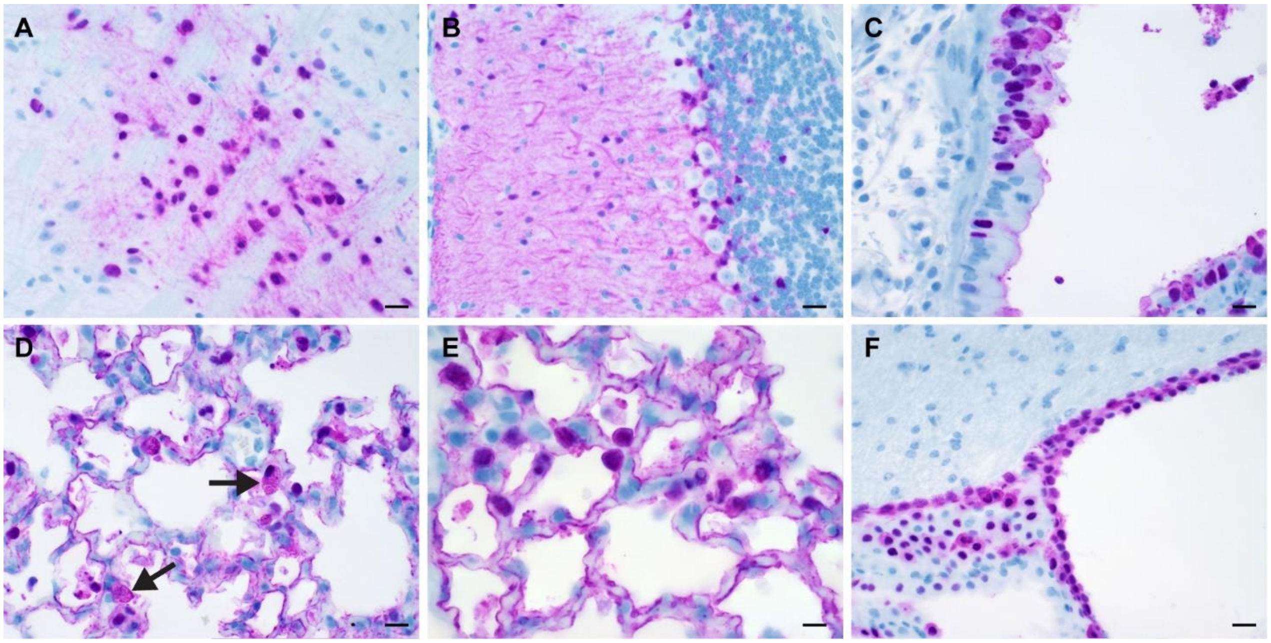

Supplementary Figure 1: Immunohistochemistry detecting influenza nucleoprotein in brain and lung of infected mice. (A) Immunoprecipitation within neurons and microglia within the midbrain (400x, scale bar = 50µm). (**B**) Immunoprecipitation within neurons and axonal processes within the cerebellum (400x, scale bar = 50µm). (**C**) Immunoprecipitation within bronchiolar epithelial cells (400x, scale bar = 50µm). (**D**) Immunoprecipitation within type I pneumocytes, alveolar cellular debris and foamy alveolar macrophages (arrow, 600x, scale bar = 20µm). (**E**) Immunoprecipitation within type I pneumocytes and prominent, plump type II pneumocytes (1000x, scale bar = 10µm). (**F**) Immunoprecipitation within ventricular epithelial (ependymal) cells (400x, scale bar = 50µm).

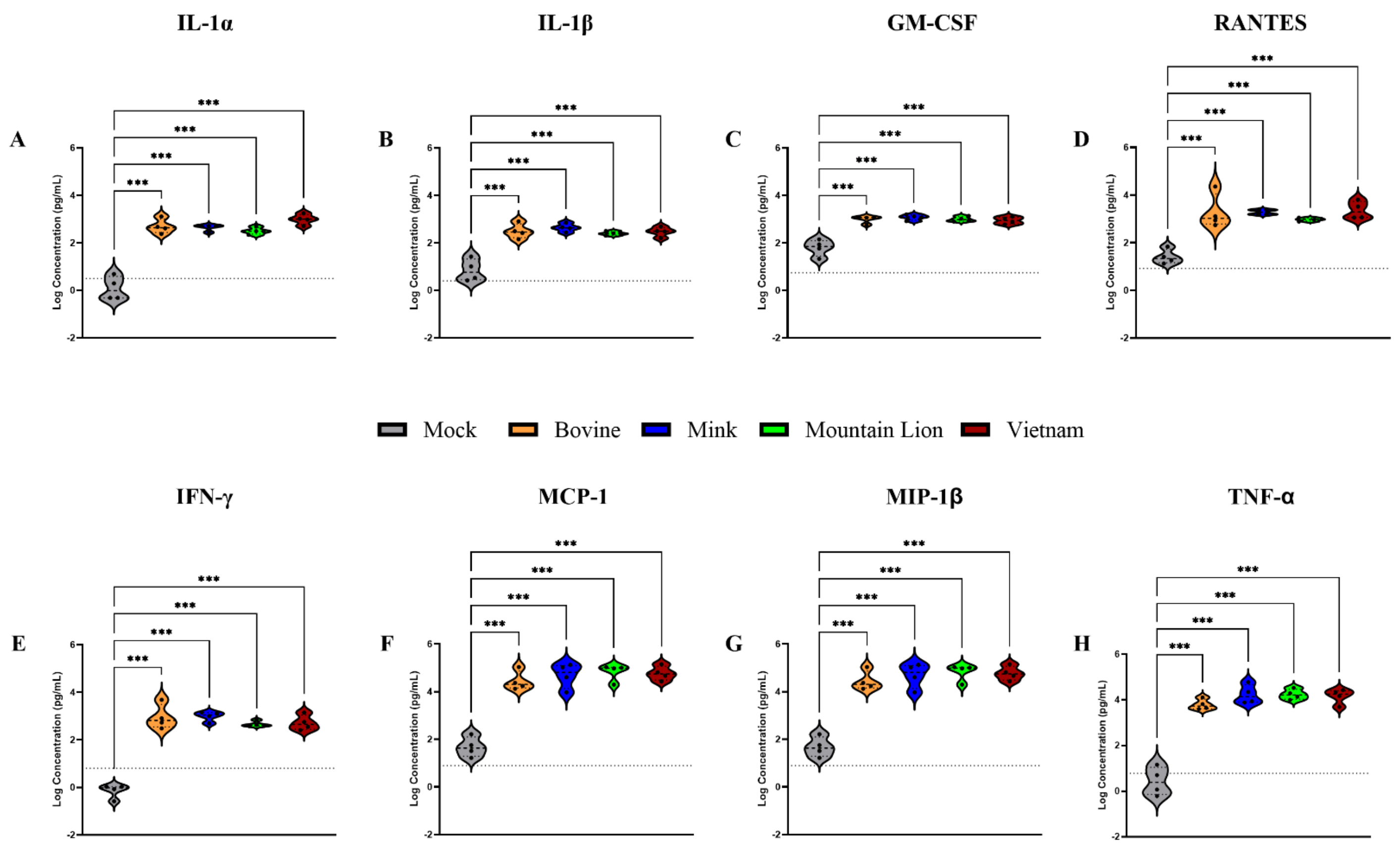

Supplementary Figure 2. Cytokine levels in the lungs of intranasally inoculated mice. Six-week-old C57BL/6J mice (n = 10 per group) inoculated orogastric with 10^5 $TCID_{50}$ of bovine, mink, mountain lion or VN1203 isolates. (**A-H**) A subset of animals (n = 4) were euthanized at day 4 for cytokine analysis following orogastric inoculations. Dashed line indicates limit of detection. Statistical analyses were performed using one-way ANOVA with Tukey's multiple comparison. *p < 0.05, **p < 0.01, ****p < 0.0001. Comparisons with p values > 0.05 are not displayed. Violin plots show interquartile range, median and density curve.

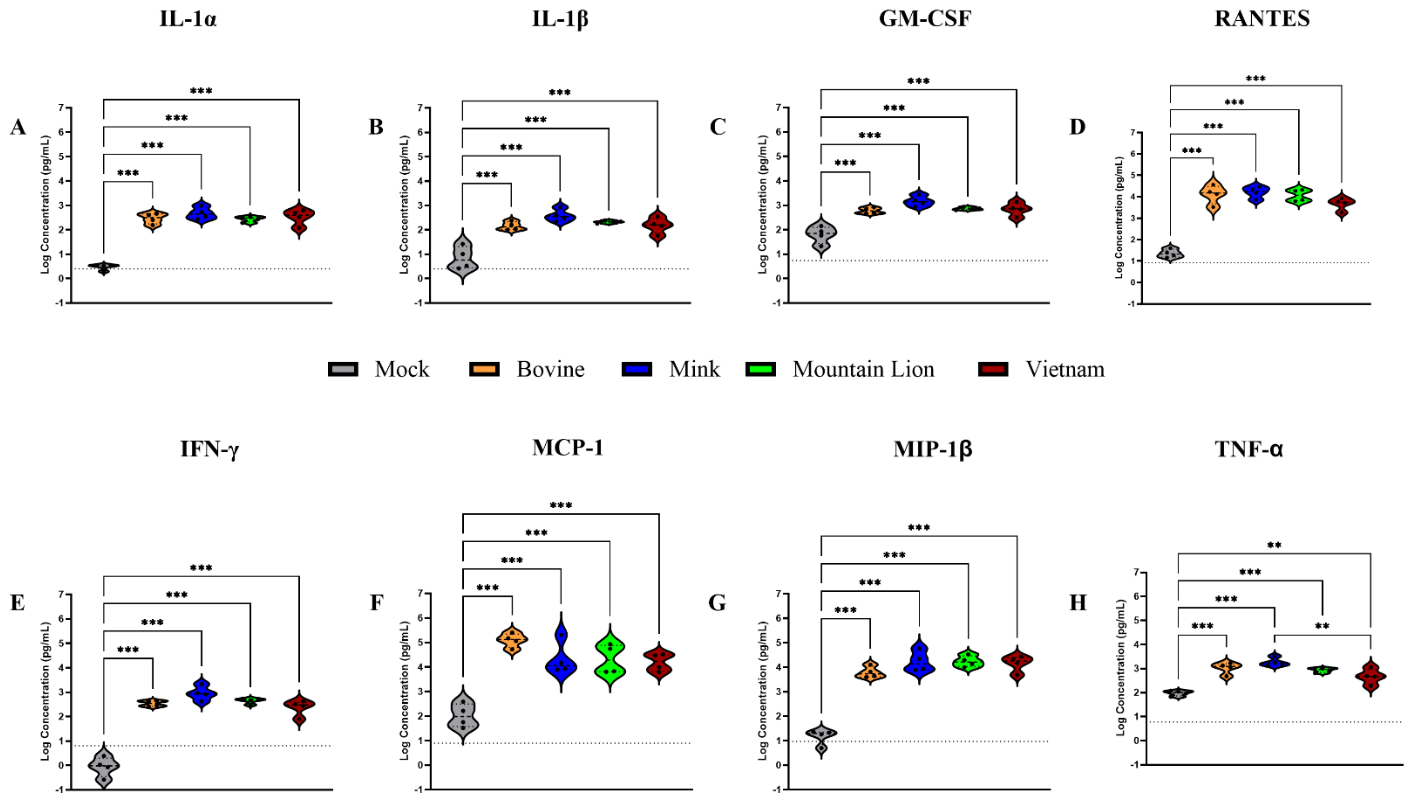

Supplementary Figure 3. Cytokine levels in the lungs of orogastric inoculated mice. Six-week-old C57BL/6J mice (n = 10 per group) inoculated orogastric with 10^5 $TCID_{50}$ of bovine, mink, mountain lion or VN1203 isolates. (**A-H**) A subset of animals (n = 4) were euthanized at day 4 for cytokine analysis following orogastric inoculations. Dashed line indicates limit of detection. Statistical analyses were performed using one-way ANOVA with Tukey's multiple comparison. *p < 0.05, **p < 0.01, ****p < 0.0001. Comparisons with p values > 0.05 are not displayed. Violin plots show interquartile range, median and density curve.

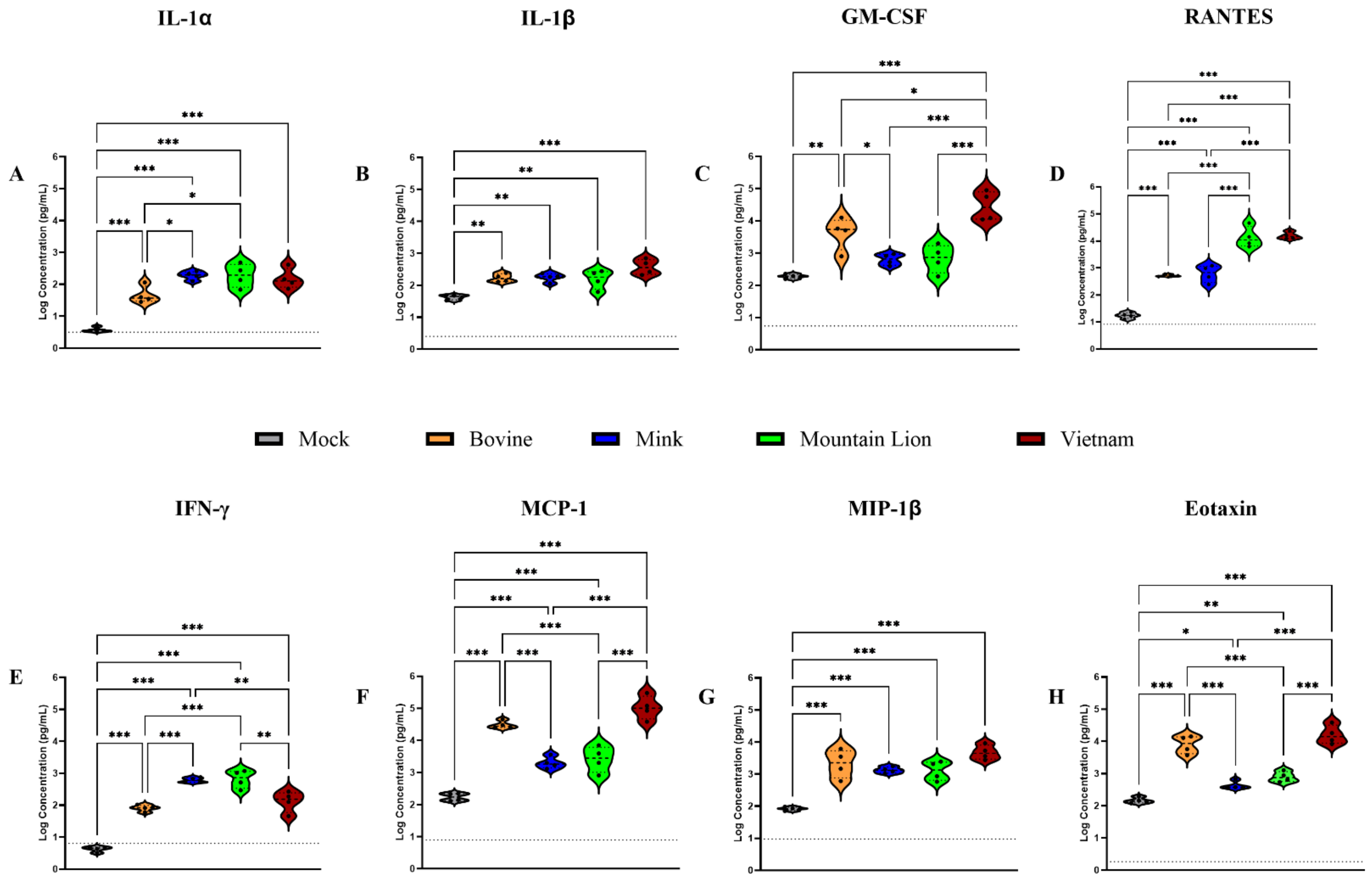

Supplementary Figure 4. Cytokine levels in the brain of orogastric inoculated mice. Six-week-old C57BL/6J mice (n = 10 per group) inoculated orogastric with 10^5 TCID$_{50}$ of bovine, mink, mountain lion or VN1203 isolates. (**A-H**) A subset of animals (n = 4) were euthanized at day 4 for cytokine analysis following orogastric inoculations. Dashed line indicates limit of detection. Statistical analyses were performed using one-way ANOVA with Tukey's multiple comparison. *p < 0.05, **p < 0.01, ****p < 0.0001. Comparisons with p values > 0.05 are not displayed. Violin plots show interquartile range, median and density curve.

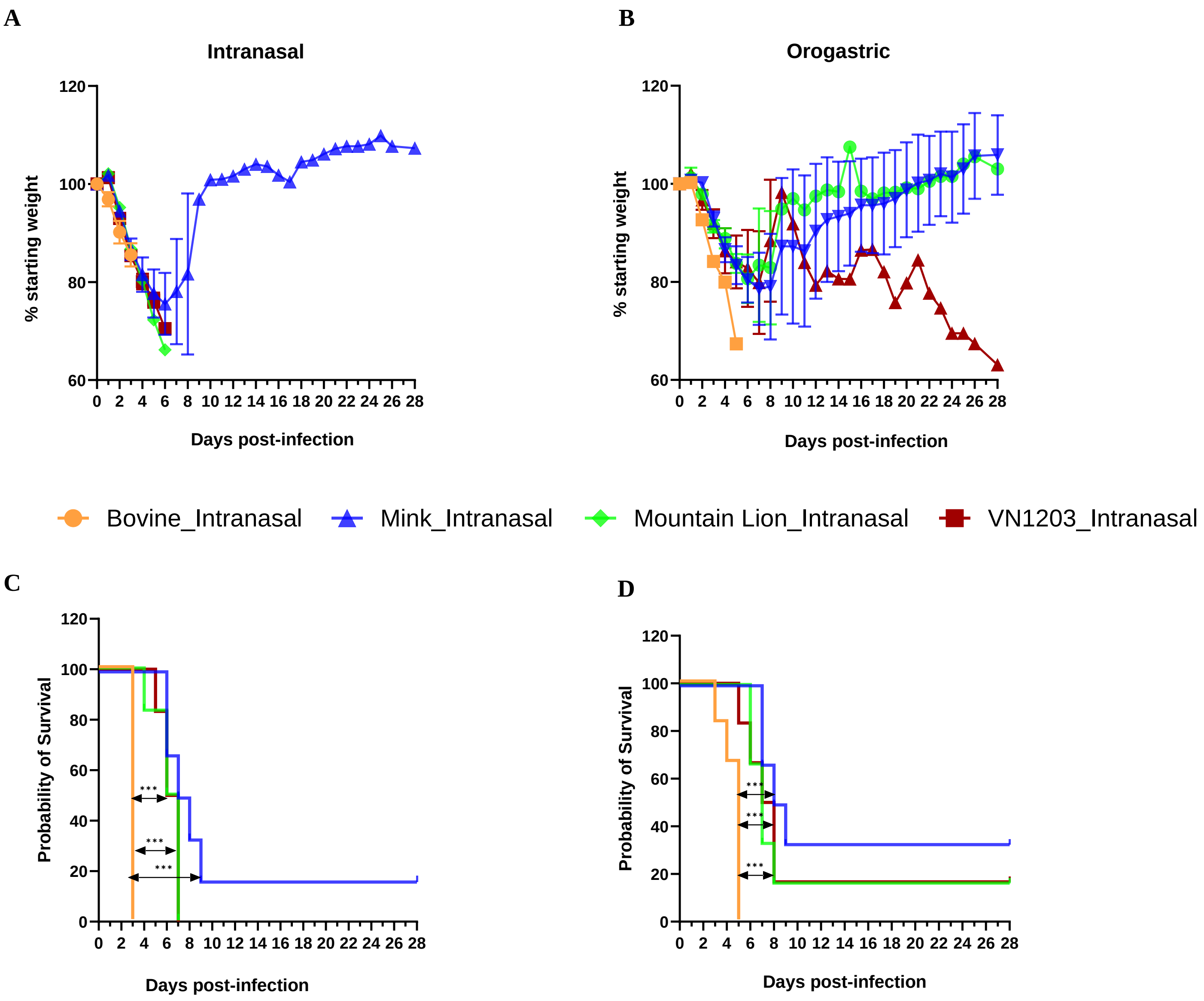

Supplementary Figure 5: **Disease progression following HPAI A(H5N1) infection.** Six-week-old BALB/c mice (n = 10 per group) were inoculated either intranasally or orogastric with 10^5 $TCID_{50}$ of bovine, mink, mountain lion and VN1203 HPAI A (H5N1) virus isolates. Mice were monitored daily for clinical signs of disease and survival. (**A**) Weight loss following intranasal inoculation. (**B**) Weight loss following orogastric inoculation. (**C**) Survival following intranasal inoculation. (**D**) Survival following orogastric inoculation. Note, animal group sizes change over time due to animals succumbing to infection. Survival proportions were calculated using the Log-rank (Mantel-Cox) test. *p < 0.05, **p < 0.01, ****p < 0.0001. Comparisons with p values > 0.05 are not displayed. (**A, B**) Data shown as mean plus standard error of the mean.

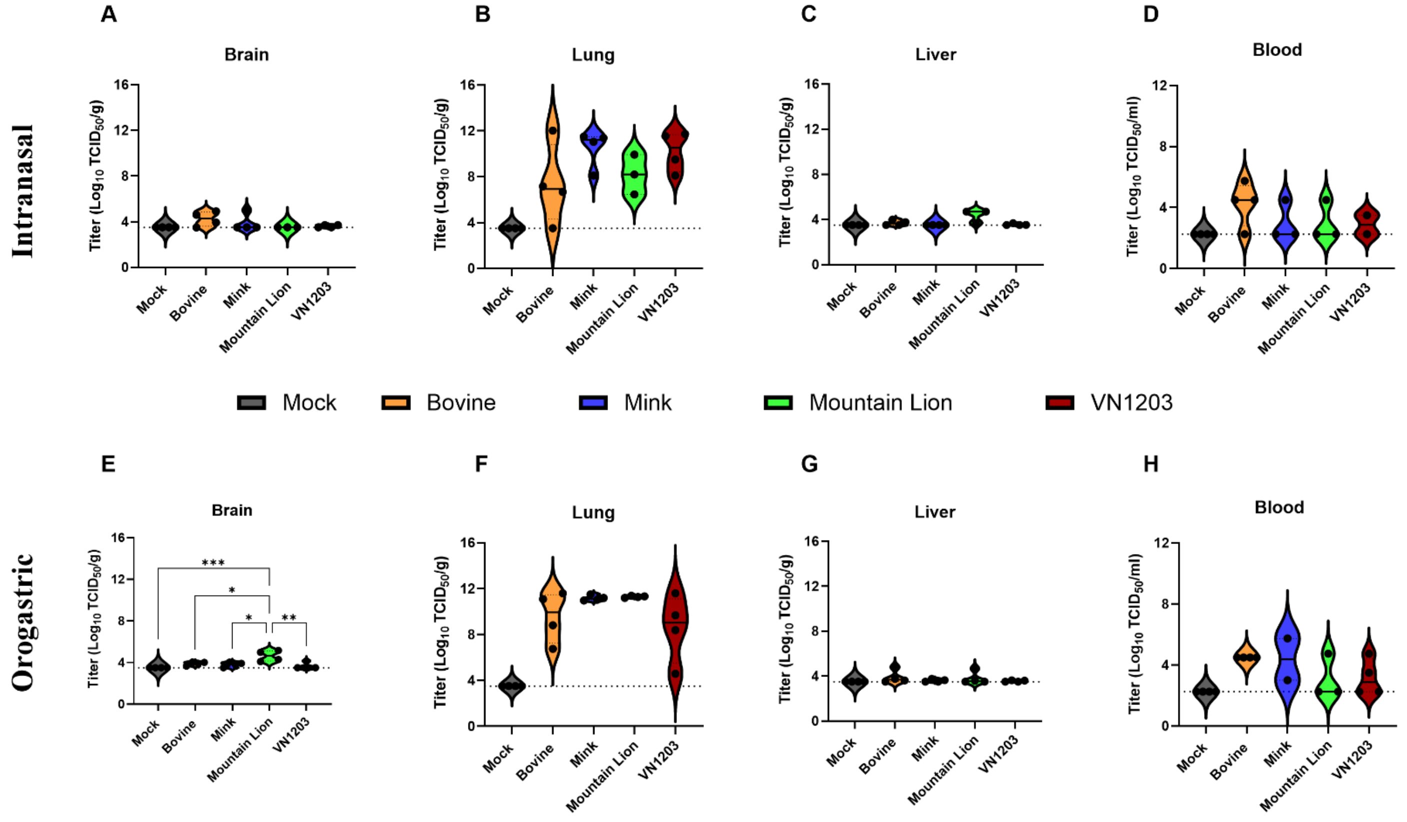

Supplementary Figure 6: **Infectious virus titers in organs in intranasally or orogastric inoculated BALB/c mice.** Six-week-old BALB/c mice (n = 10 per group) were inoculated either intranasally or orogastric with 10^5 $TCID_{50}$ of bovine, mink, mountain lion and VN1203 isolates. A subset of animals (n = 4) were euthanized at day 2 for evaluation of replicating virus, intranasal (**A-D**) or orogastric inoculations (**E-H**). Fewer than 4 blood samples from mice infected with mink, mountain lion and VN1203 (intranasally inoculated) isolates were available for analysis. Dashed line indicates limit of detection. Statistical analyses were performed using two-way ANOVA with Tukey's multiple comparison. *p < 0.05, **p < 0.01, ****p < 0.0001. Comparisons with p values > 0.05 are not displayed. Violin plots show interquartile range, median and density curve.

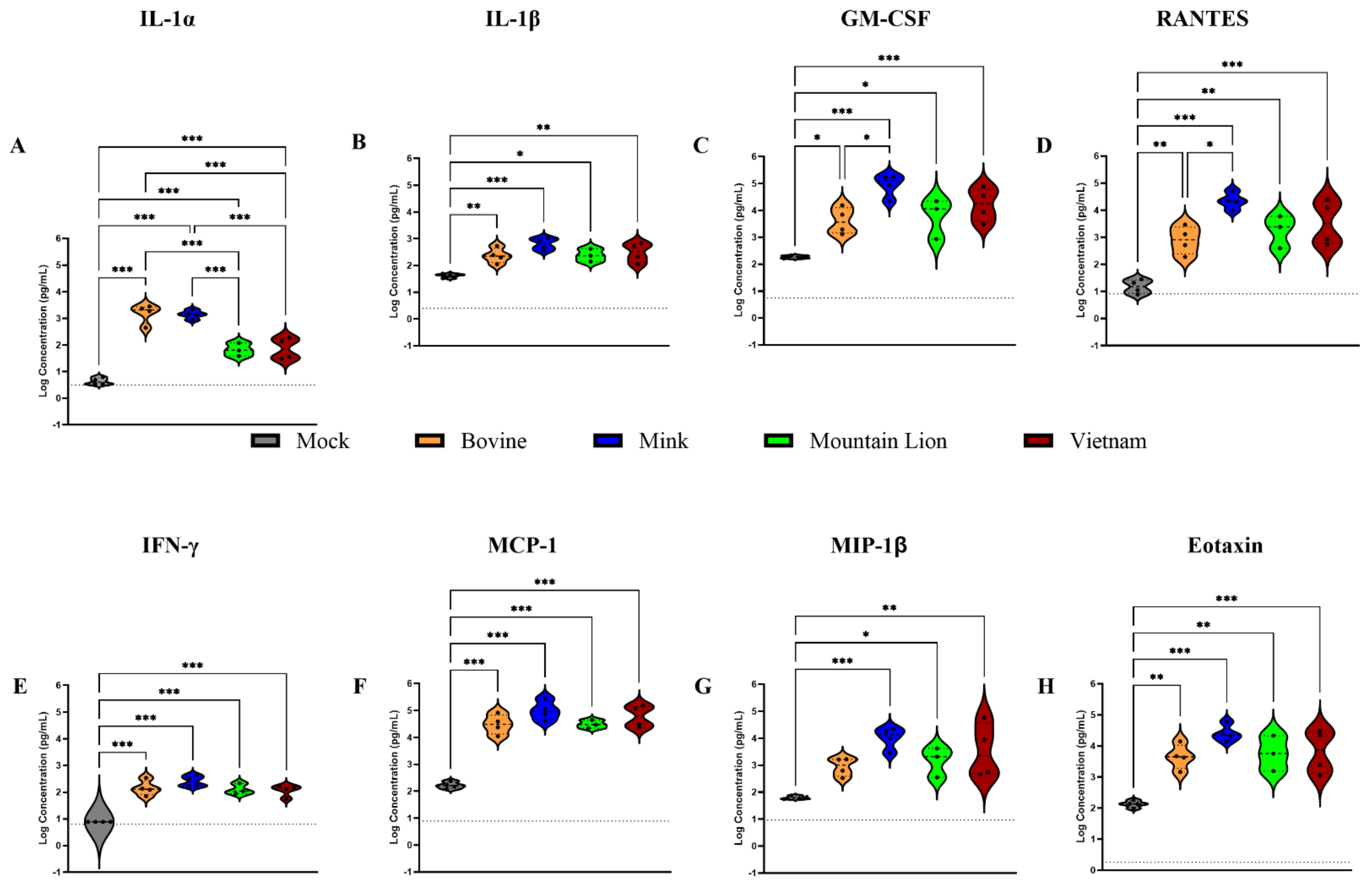

Supplementary Figure 7. Brain tissue cytokine induction by H5N1 viruses intranasally inoculated mice. Six-week-old BALB/c mice (n = 10 per group) were inoculated intranasally with 10^5 $TCID_{50}$ of bovine, mink, mountain lion and VN1203 isolates. (**A-H**) A subset of animals (n = 4) were euthanized at day 2 for evaluation of cytokine levels. One animal in the mountain lion group succumbed during viral challenge at day 0 (cause unknown) leaving 3 animals in the group. Dashed line indicates limit of detection. Statistical analyses were performed using two-way ANOVA with Tukey's multiple comparison. $*p < 0.05$, $**p < 0.01$, $****p < 0.0001$. Comparisons with p values > 0.05 are not displayed. Violin plots show interquartile range, median and density curve.

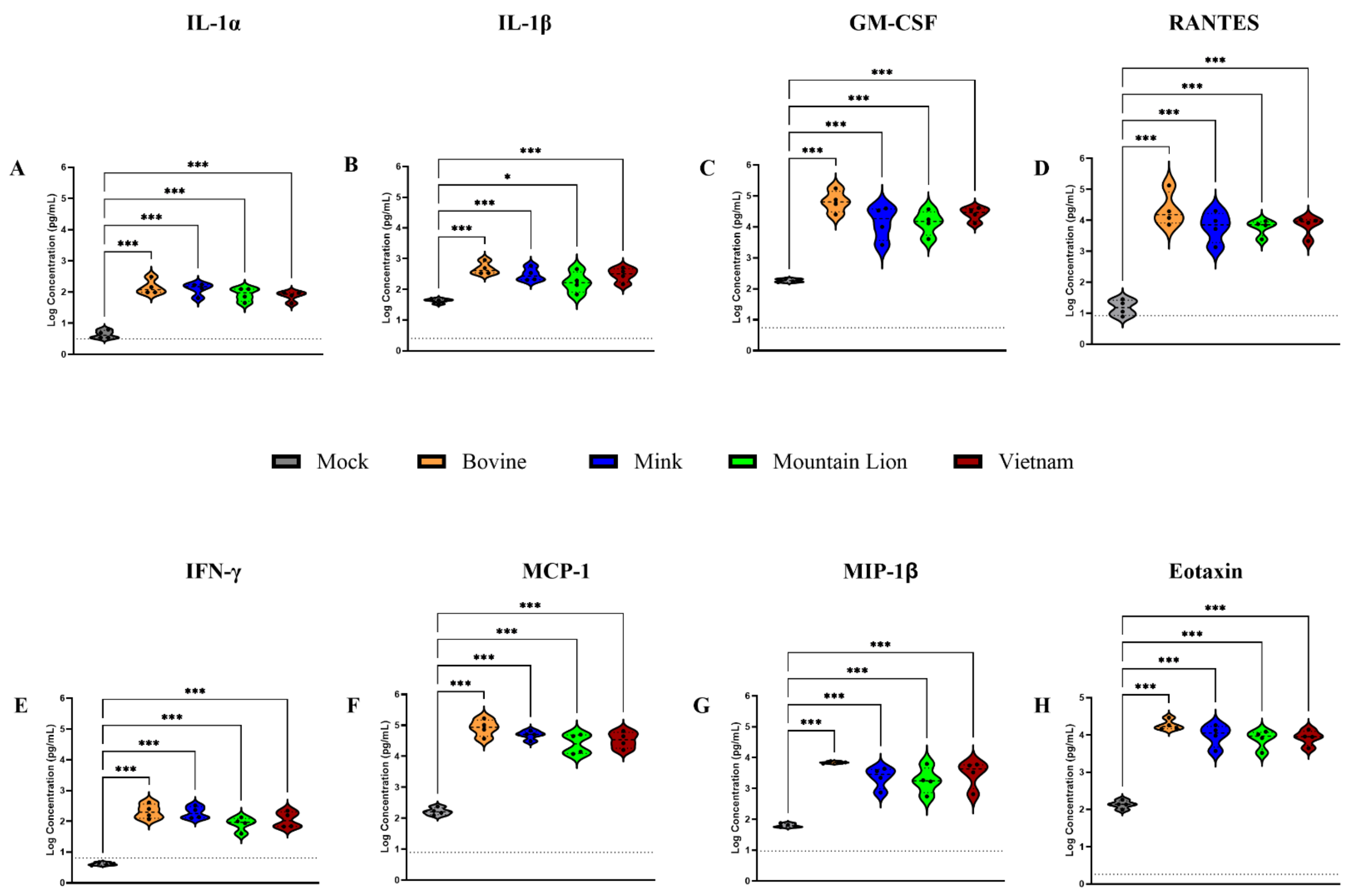

Supplementary Figure 8. Brain tissue cytokine induction by H5N1 viruses in orogastric inoculated mice. Six-week-old BALB/c mice (n = 10 per group) were inoculated intranasally with 10^5 TCID$_{50}$ of bovine, mink, mountain lion and VN1203 isolates. (**A-H**) A subset of animals (n = 4) were euthanized at day 2 for evaluation of cytokine levels. Dashed line indicates limit of detection. Statistical analyses were performed using two-way ANOVA with Tukey's multiple comparison. *p < 0.05, **p < 0.01, ****p < 0.0001. Comparisons with p values > 0.05 are not displayed. Violin plots show interquartile range, median and density curve.

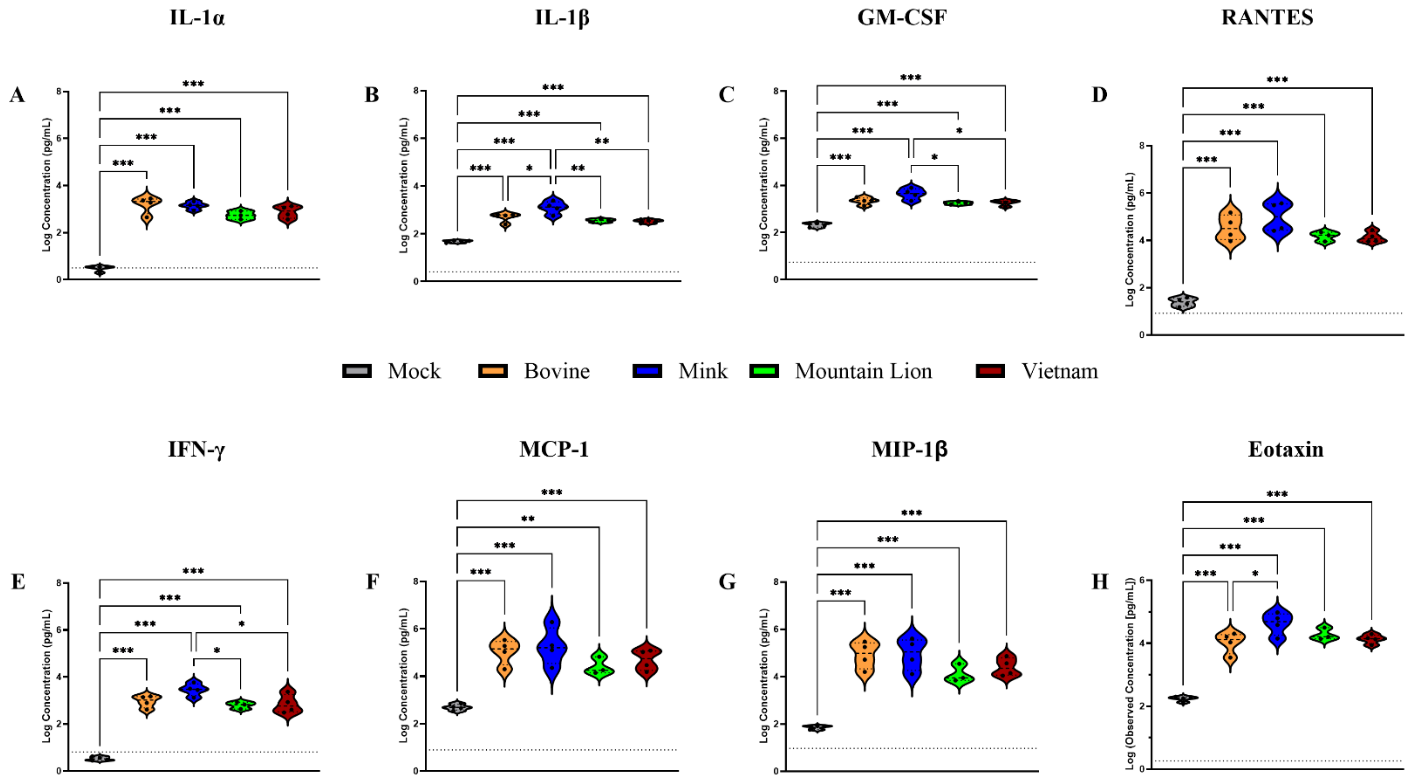

Supplementary Figure 9. Lung tissue cytokine induction by H5N1 viruses in intranasally inoculated mice. Six-week-old BALB/c mice (n = 10 per group) were inoculated intranasally with 10^5 $TCID_{50}$ of bovine, mink, mountain lion and VN1203 isolates. (**A-H**) A subset of animals (n = 4) were euthanized at day 2 for evaluation of cytokine levels. One animal in the mountain lion group succumbed during viral challenge at day 0 (cause unknown) leaving 3 animals in the group. Dashed line indicates limit of detection. Statistical analyses were performed using two-way ANOVA with Tukey's multiple comparison. *p < 0.05, **p < 0.01, ****p < 0.0001. Comparisons with p values > 0.05 are not displayed. Violin plot shows interquartile range, median and density curve. Violin plots show interquartile range, median and density curve.

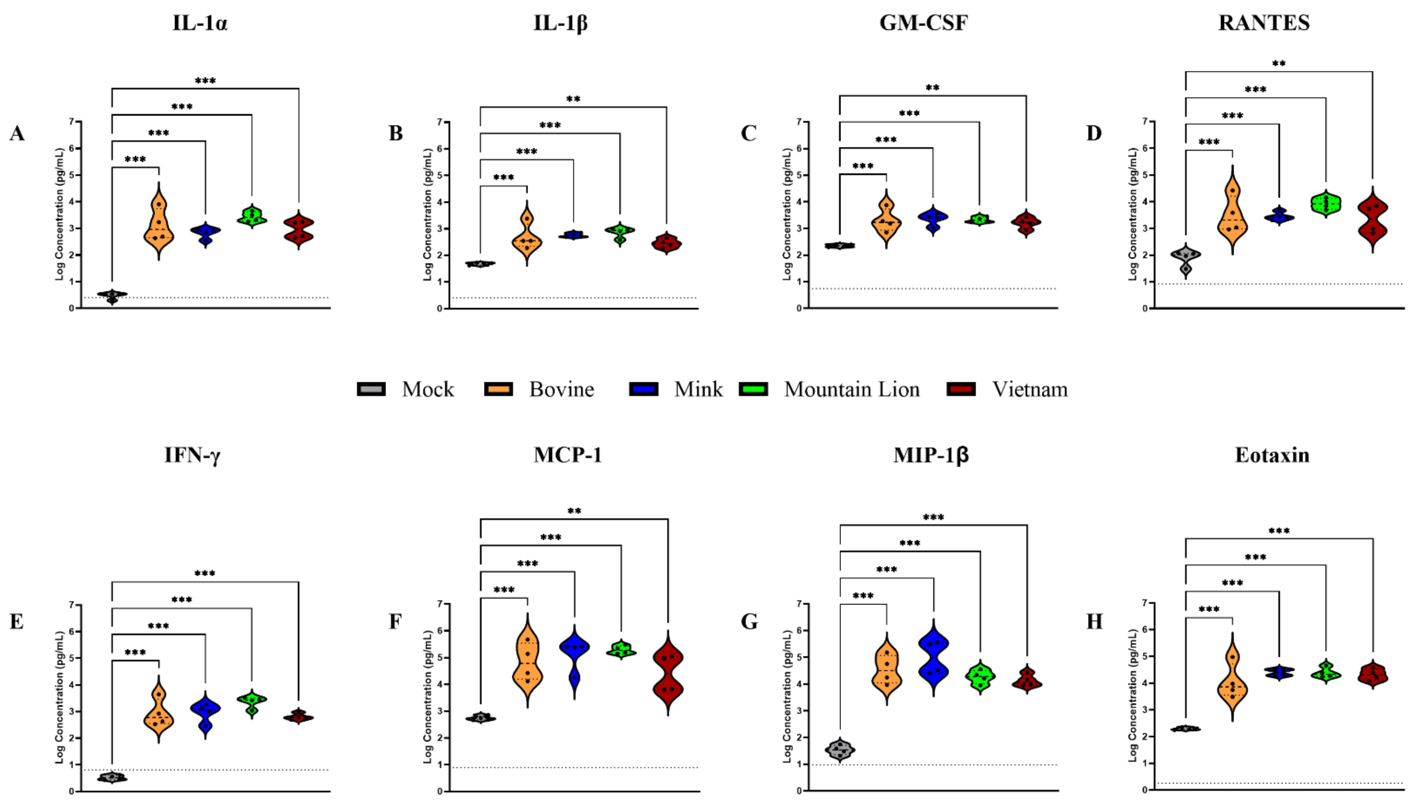

Supplementary Figure 10. Lung tissue cytokine induction by H5N1 viruses in orogastric inoculated mice. Six-week-old BALB/c mice (n = 10 per group) were inoculated intranasally with 10^5 $TCID_{50}$ of bovine, mink, mountain lion and VN1203 isolates. (**A-H**) A subset of animals (n = 4) were euthanized at day 2 for evaluation of cytokine levels. Dashed line indicates limit of detection. Statistical analyses were performed using two-way ANOVA with Tukey’s multiple comparison. $*p < 0.05$, $**p < 0.01$, $****p < 0.0001$. Comparisons with p values > 0.05 are not displayed. Violin plots show interquartile range, median and density curve.

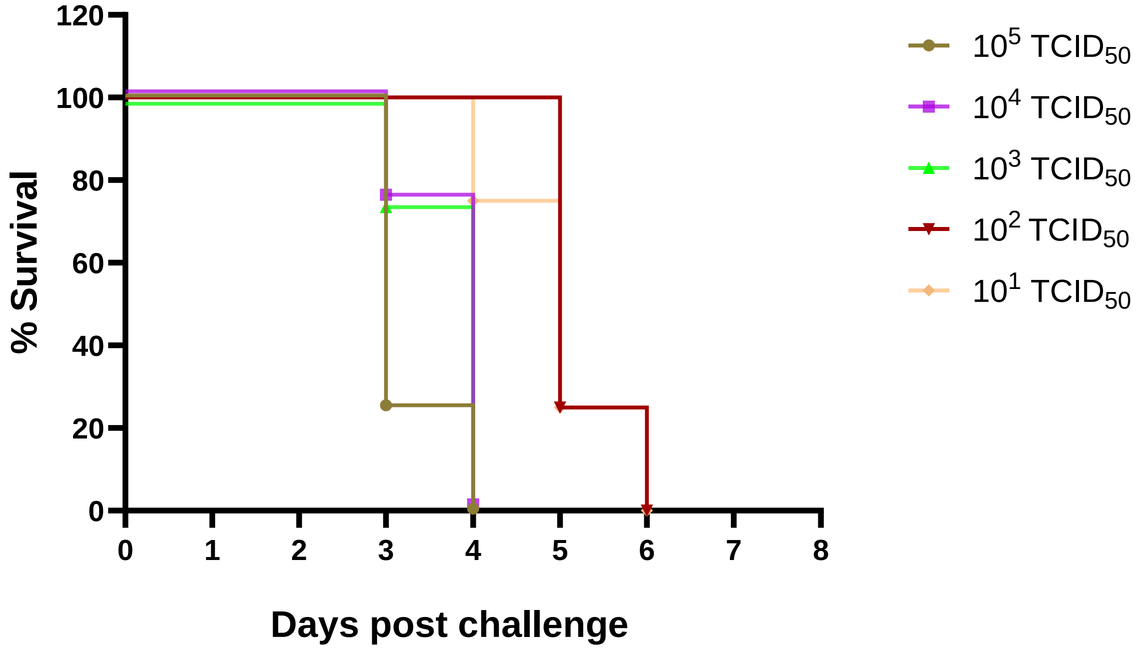

Supplementary Figure 11. Pathogenicity in mice after intranasally inoculation of the bovine H5N1 virus. Mice (n = 4 per group) were challenged intranasally with indicated doses of the bovine isolate and monitored for survival.

Supplementary Table 1. Amino acid identity of the mountain lion, mink and VN1204 compared to the bovine isolate

Genome Segment	Protein(s)	A/mountain lion/MT/1/2024) (%)	A/mink/Spain/3691-8_22VIR10586-10/2022 (%)	A/Vietnam/1203/04 (%)
**1**	PB2	97.76	98.42	97.89
**2**	PB1	99.74	98.94	98.41
	PB1-F2	94.44	74.44	81.11
**3**	PA	99.72	97.77	97.63
	PA-X	99.60	97.23	96.84
**4**	HA	99.82	98.94	91.53
**5**	NP	99.00	97.79	99.20
**6**	NA	99.57	97.01	93.10
**7**	M1	100.00	97.22	91.25
	M2	100.00	96.91	30.49
**8**	NS1	98.70	90.87	91.96
	NS2/NEP	98.99	90.91	91.92

Supplementary Table 2. Compared to Bovine (PB2) Segment 1

Bovine		VN1203	Mountain Lion	Mink
**AA**	Position			
**A**	58	T	T	T
**I**	109	V	V	V
**I**	139	V	I	V
**H**	233	P	H	H
**R**	251	K	K	R
**R**	340	K	R	R
**K**	353	R	R	K
**G**	362	E	E	E
**E**	391	Q	E	E
**N**	441	D	D	D
**I**	451	I	I	V
**S**	489	S	P	S
**I**	495	V	V	V
**L**	607	L	I	L
**I**	615	I	M	I
**E**	627	K	E	E
**L**	631	M	M	M
**I**	649	V	V	V
**K**	663	K	R	K
**V**	667	V	I	V
**A**	676	T	T	T
**K**	677	E	E	E
**G**	682	G	S	G
**G**	685	G	G	R

AA- amino acid

Supplementary Table 3. Compared to Bovine (PB1) Segment 2

Bovine		VN1203	Mountain Lion	Mink
**AA**	Position			
**N**	16	N	N	D
**S**	59	S	S	T
**D**	75	E	D	E
**E**	90	E	E	E
**V**	113	I	V	V
**V**	149	I	V	V
**I**	179	M	I	I
**R**	215	K	R	R
**S**	375	N	S	N
**S**	384	L	S	S
**R**	386	K	R	R
**K**	388	K	K	R
**I**	392	I	I	I
**K**	430	R	K	K
**D**	464	D	N	D
**V**	527	V	V	I
**P**	587	A	P	A

AA- amino acid

Supplementary Table 4. Compared to Bovine (PA) Segment 3

Bovine		VN1203	Mountain Lion	Mink
**AA**	Position			
**G**	58	S	G	G
**M**	61	I	M	I
**A**	70	A	A	V
**A**	85	T	A	T
**M**	86	M	M	T
**D**	101	D	D	E
**R**	113	K	R	K
**I**	129	T	I	I
**K**	142	D	K	K
**R**	204	K	R	R
**I**	219	L	L	L
**P**	277	S	P	S
**A**	343	A	A	S
**I**	348	I	I	L
**N**	350	N	N	S
**E**	352	E	E	D
**S**	421	I	S	S
**V**	441	M	V	M
**R**	497	K	K	K
**I**	554	V	I	I
**L**	558	S	L	S
**S**	608	T	S	T
**V**	669	A	V	V
**T**	712	A	T	T

AA- amino acid

Supplementary Table 5. Compared to Bovine (HA) Segment 4

Bovine		VN1203	Mountain Lion	Mink
**AA**	Position			
**N**	3	K	N	N
**L**	8	F	L	L
**T**	52	K	T	T
**N**	61	D	N	N
**K**	69	R	K	K
**R**	88	N	R	R
**R**	98	K	R	R
**A**	102	V	A	A
**S**	110	D	S	S
**L**	111	F	L	L
**M**	120	L	M	L
**Q**	131	Q	Q	L
**P**	139	S	P	P
**N**	140	S	N	N
**T**	143	A	T	T
**A**	149	S	A	A
**A**	156	K	A	A
**P**	157	S	P	P
**D**	171	S	D	D
**A**	172	T	A	A
**I**	178	R	I	I
**R**	185	Q	R	R
**I**	190	V	I	I
**S**	197	P	S	S
**R**	199	D	N	N
**N**	205	K	N	N
**K**	208	Q	K	K
**I**	211	T	I	T
**A**	226	V	A	V
**K**	228	R	K	K
**Q**	234	K	Q	Q
**R**	239	S	R	R
**D**	243	E	D	D
**D**	252	N	D	D
**H**	256	N	H	H
**G**	284	E	G	G
**V**	285	L	V	V
**H**	289	N	H	H
**V**	298	M	V	V
**K**	326	R	K	K
**L**	338	Q	L	L
**K**	341	R	K	K
**Deletion**	345	K	Del	Del
**I**	352	I	I	I

**I**	391	I	I	M
**V**	527	I	V	V
**T**	529	I	T	T
**A**	539	V	A	A
**M**	549	V	M	M

AA- amino acid

Supplementary Table 6. Compared to Bovine (NP) Segment 5

Bovine		VN1203	Mountain Lion	Mink
**AA**	Position			
**V**	33	V	V	I
**G**	34	S	G	G
**H**	52	Y	Y	Y
**K**	77	R	K	K
**M**	105	V	V	V
**A**	129	A	A	S
**L**	193	L	L	I
**I**	201	I	I	V
**F**	230	F	L	F
**S**	269	S	S	A
**T**	433	T	N	T
**N**	482	N	S	S
**N**	483	N	N	K
**Y**	496	Y	Y	F
**N**	498	N	N	S

AA- amino acid

Supplementary Table 7. Compared to Bovine (NA) Segment 6

Bovine		VN1203	Mountain Lion	Mink
**AA**	Position			
**K**	6	K	K	R
**T**	8	T	T	I
**I**	10	I	I	T
**I**	17	T	I	I
**I**	29	M	I	I
**Q**	39	H	Q	Q
**Y**	44	H	Y	Y
**P**	46	S	P	P
**C**	49	Deletion	C	C
**N**	50	Deletion	N	N
**Q**	51	Deletion	Q	Q
**S**	52	Deletion	S	S
**I**	53	Deletion	I	I
**I**	54	Deletion	I	I
**T**	55	Deletion	T	T
**Y**	56	Deletion	Y	Y
**E**	57	Deletion	E	E
**N**	58	Deletion	N	N
**N**	59	Deletion	N	N
**T**	60	Deletion	T	T
**W**	61	Deletion	W	W
**V**	62	Deletion	V	V
**N**	63	Deletion	N	N
**Q**	64	Deletion	Q	Q
**T**	65	Deletion	T	T
**Y**	66	Deletion	Y	Y
**I**	67	Deletion	I	V
**N**	68	Deletion	N	N
**F**	74	F	F	S
**A**	76	T	A	A
**Q**	78	K	Q	Q
**T**	81	A	T	T
**T**	83	K	T	T
**S**	95	N	S	S
**I**	99	V	I	I
**G**	105	S	G	G
**Y**	155	H	Y	Y
**V**	163	V	V	L
**I**	188	T	I	I
**Y**	253	H	Y	Y
**I**	258	M	I	I
**M**	269	L	M	L
**N**	270	D	N	N
**D**	284	N	D	D

**N**	287	E	N	E
**M**	289	T	M	M
**I**	321	V	I	V
**S**	336	G	S	S
**M**	338	V	M	M
**P**	339	S	P	S
**S**	366	N	S	S
**S**	369	S	S	I
**E**	382	E	N	E
**V**	389	V	M	V
**E**	395	E	E	A
**I**	396	I	I	M
**S**	405	S	S	T
**M**	418	I	M	M
**N**	434	S	N	N

AA- amino acid

Supplementary Table 8. Compared to Bovine (M2 and M2) Segment 7

Bovine		VN1203	Mountain Lion	Mink
**AA**	Position			
**V**	15	I	V	V
**R**	27	K	R	R
**L**	55	L	L	M
**S**	82	N	S	N
**S**	85	N	S	N
**T**	87	N	T	N
**R**	101	K	R	K
**A**	140	T	A	A
**L**	144	F	L	L
**L**	165	M	L	L
**T**	168	I	T	T
**V**	200	A	V	A
**V**	205	I	V	V
**S**	207	N	S	S
**S**	224	N	S	S
**T**	227	A	T	A
**D**	232	N	D	D
**L**	248	M	L	L
**S**	254	Y	S	S
**R**	255	C	R	R
**Y**	256	C	Y	Y
**P**	273	S	P	P
**R**	278	H	R	R
**R**	287	K	R	K
**F**	290	C	F	F
**S**	294	G	S	S
**H**	297	Y	H	H
**Q**	312	Q	Q	R

AA- amino acid

Supplementary Table 9. Compared to Bovine (NS1 & NEP) Segment 8

Bovine		VN1203	Mountain Lion	Mink
**AA**	Position			
**L**	7	S	S	S
**E**	26	E	E	D
**L**	27	L	L	M
**Q**	40	Q	Q	Q
**G**	47	G	G	S
**S**	48	N	S	S
**L**	52	L	L	I
**T**	56	T	T	A
**A**	60	A	A	S
**V**	65	V	V	I
**E**	70	E	E	G
**E**	71	G	E	E
**E**	75	K	E	E
**T**	80	Deletion	T	T
**I**	81	Deletion	I	I
**A**	82	Deletion	A	A
**S**	83	Deletion	S	S
**V**	84	Deletion	V	V
**P**	85	P	S	P
**P**	87	S	P	P
**S**	116	C	S	S
**R**	118	K	R	R
**N**	127	T	N	N
**N**	139	D	N	N
**P**	164	P	P	P
**D**	171	G	D	S
**V**	194	V	V	L
**S**	195	T	S	S
**T**	197	T	T	N
**L**	198	I	L	L
**R**	204	R	R	G
**S**	205	N	S	S
**N**	207	D	N	N
**D**	209	D	D	N
**P**	212	L	P	P
**L**	214	L	L	F
**P**	215	P	P	T
**K**	217	N	K	E
**R**	220	R	R	Q
**K**	221	*	K	K
**M**	222		M	L
**E**	223		E	A

*Stop codon, AA- amino acid
