# Supplementary material for: Highly pathogenic avian influenza H5N1 clade 2.3.4.4b genotype B3.13 is highly virulent for mice, rapidly causing acute pulmonary and neurologic disease
Source: Nat Commun. 2025 Jul 1;16:5738. doi: 10.1038/s41467-025-60407-y (PMC12216816; doi:10.1038/s41467-025-60407-y)
Supplement: Supplementary file 2 — Description of Additional Supplementary Files [file 41467_2025_60407_MOESM2_ESM.pdf]

Description of Additional Supplementary Files

Supplementary data 1: Histopathology matrix in C57BL/6J mice. Histology scores and immunohistochemistry results for C57BL/6J mice following oral and intranasal inoculation with HPAI A(H5N1)

Supplementary data 2: Histopathology matrix in BALB/c mice. Histology scores and immunohistochemistry results for BALB/c mice following oral and intranasal inoculation with HPAI A(H5N1)

Supplementary data 3: **Histopathology matrix in intranasally inoculated C57BL/6J and BALB/c mice.** Histology scores and immunohistochemistry results for C57BL/6J and BALB/c mice following intranasal inoculation with HPAI A(H5N1)
