# Supplementary material for: Highly pathogenic avian influenza H5N1 clade 2.3.4.4b genotype B3.13 is highly virulent for mice, rapidly causing acute pulmonary and neurologic disease
Source: Nat Commun. 2025 Jul 1;16:5738. doi: 10.1038/s41467-025-60407-y (PMC12216816; doi:10.1038/s41467-025-60407-y)
Supplement: Supplementary file 6 — Reporting Summary [file 41467_2025_60407_MOESM6_ESM.pdf]

nature portfolio

Corresponding author(s): Heinz Feldman
Last updated by author(s): Mar 12, 2025

Reporting Summary

Nature Portfolio wishes to improve the reproducibility of the work that we publish. This form provides structure for consistency and transparency in reporting. For further information on Nature Portfolio policies, see our Editorial Policies and the Editorial Policy Checklist.

Statistics

For all statistical analyses, confirm that the following items are present in the figure legend, table legend, main text, or Methods section.

n/a	Confirmed	
☐	☒	The exact sample size (n) for each experimental group/condition, given as a discrete number and unit of measurement
☒	☐	A statement on whether measurements were taken from distinct samples or whether the same sample was measured repeatedly
☐	☒	The statistical test(s) used AND whether they are one- or two-sided<br>*Only common tests should be described solely by name; describe more complex techniques in the Methods section.*
☒	☐	A description of all covariates tested
☒	☐	A description of any assumptions or corrections, such as tests of normality and adjustment for multiple comparisons
☒	☐	A full description of the statistical parameters including central tendency (e.g. means) or other basic estimates (e.g. regression coefficient) AND variation (e.g. standard deviation) or associated estimates of uncertainty (e.g. confidence intervals)
☐	☒	For null hypothesis testing, the test statistic (e.g. F, t, r) with confidence intervals, effect sizes, degrees of freedom and P value noted<br>*Give P values as exact values whenever suitable.*
☒	☐	For Bayesian analysis, information on the choice of priors and Markov chain Monte Carlo settings
☒	☐	For hierarchical and complex designs, identification of the appropriate level for tests and full reporting of outcomes
☒	☐	Estimates of effect sizes (e.g. Cohen's d, Pearson's r), indicating how they were calculated

Our web collection on statistics for biologists contains articles on many of the points above.

Software and code

Policy information about availability of computer code

Data collection	No software was used for data collection
Data analysis	Prism 10 (GraphPad), Bowtie2 v.2.2.928, Picard MarkDuplicates v.2.26.9, GATK HaplotypeCaller v.4.1.2.029

For manuscripts utilizing custom algorithms or software that are central to the research but not yet described in published literature, software must be made available to editors and reviewers. We strongly encourage code deposition in a community repository (e.g. GitHub). See the Nature Portfolio guidelines for submitting code & software for further information.

Data

Policy information about availability of data

All manuscripts must include a data availability statement. This statement should provide the following information, where applicable:

- Accession codes, unique identifiers, or web links for publicly available datasets
- A description of any restrictions on data availability
- For clinical datasets or third party data, please ensure that the statement adheres to our policy

All relevant data are within the manuscript and its Supporting Information files.

Research involving human participants, their data, or biological material

Policy information about studies with human participants or human data. See also policy information about sex, gender (identity/presentation), and sexual orientation and race, ethnicity and racism.

Reporting on sex and gender	N/A
Reporting on race, ethnicity, or other socially relevant groupings	N/A
Population characteristics	N/A
Recruitment	N/A
Ethics oversight	N/A

Note that full information on the approval of the study protocol must also be provided in the manuscript.

Field-specific reporting

Please select the one below that is the best fit for your research. If you are not sure, read the appropriate sections before making your selection.

☒ Life sciences ☐ Behavioural & social sciences ☐ Ecological, evolutionary & environmental sciences

For a reference copy of the document with all sections, see nature.com/documents/nr-reporting-summary-flat.pdf

Life sciences study design

All studies must disclose on these points even when the disclosure is negative.

Sample size	Survival: Even if only 20% animals survive or show no disease in a low dose group (10^3 TCID50) compared to 100% lethality or severe disease in a higher dose group (10^5 TCID50) we need 6 animals per group. Pearson's Chi-squared test (pooled variance).<br>Cytokines and viral titers: We expect to see at least 6-fold difference between the infected and mock infected animals with an estimated standard deviation of 2. Standardization of outcomes would make the mean of naïve animals 1, and increased viral titers or cytokines expression of genes of interest 6, resulting in a mean difference of 5. We need a minimum of 4 animals per group (Two-sample t-Test).
Data exclusions	No data was excluded.
Replication	Experiments were not replicates but each mouse is considered a biological replicate.
Randomization	The animals were distributed among the groups randomly.
Blinding	Downstream assays were performed by researchers blinded to the study group, and the pathologist was blinded during the study.

Reporting for specific materials, systems and methods

We require information from authors about some types of materials, experimental systems and methods used in many studies. Here, indicate whether each material, system or method listed is relevant to your study. If you are not sure if a list item applies to your research, read the appropriate section before selecting a response.

Materials & experimental systems

n/a	Involved in the study	
☐	☒	Antibodies
☐	☒	Eukaryotic cell lines
☒	☐	Palaeontology and archaeology
☐	☒	Animals and other organisms
☒	☐	Clinical data
☒	☐	Dual use research of concern
☒	☐	Plants

Methods

n/a	Involved in the study	
☒	☐	ChIP-seq
☒	☐	Flow cytometry
☒	☐	MRI-based neuroimaging

Antibodies

Antibodies used	Millipore Sigma Anti-influenza antibody (ABF1820-25UL), Roche Tissue Diagnostics DISCOVERY Omnimap anti-rabbit HRP (760-4311)
Validation	Validation of antibodies were performed in house using manufacturers validated methods on known positive tissues. Negative controls (cells, tissues, sera) that were infected with an array of different viruses were used to gauge specificity. Secondary antibodies were validated by the commercial entities.

Eukaryotic cell lines

Policy information about cell lines and Sex and Gender in Research

Cell line source(s)	Madin-Darby canine kidney (MDCK) from Dr. Ron Fouchier, Erasmus MC, Netherlands
Authentication	Not authenticated
Mycoplasma contamination	MDCK cells tested negative monthly
Commonly misidentified lines (See ICLAC register)	None

Animals and other research organisms

Policy information about studies involving animals; ARRIVE guidelines recommended for reporting animal research, and Sex and Gender in Research

Laboratory animals	Mus musculus (Mouse)
Wild animals	None
Reporting on sex	Findings apply to males and females.
Field-collected samples	None
Ethics oversight	Animal experiments were approved by the Rocky Mountain Laboratories Animal Care and Use Committee (ACUC) and carried out in an AALAC accredited facility.

Note that full information on the approval of the study protocol must also be provided in the manuscript.

Plants

Seed stocks	*Report on the source of all seed stocks or other plant material used. If applicable, state the seed stock centre and catalogue number. If plant specimens were collected from the field, describe the collection location, date and sampling procedures.*
Novel plant genotypes	*Describe the methods by which all novel plant genotypes were produced. This includes those generated by transgenic approaches, gene editing, chemical/radiation-based mutagenesis and hybridization. For transgenic lines, describe the transformation method, the number of independent lines analyzed and the generation upon which experiments were performed. For gene-edited lines, describe the editor used, the endogenous sequence targeted for editing, the targeting guide RNA sequence (if applicable) and how the editor was applied.*
Authentication	*Describe any authentication procedures for each seed stock used or novel genotype generated. Describe any experiments used to assess the effect of a mutation and, where applicable, how potential secondary effects (e.g. second site T-DNA insertions, mosiacism, off-target gene editing) were examined.*
