# Supplementary material for: Highly pathogenic avian influenza H5N1 clade 2.3.4.4b genotype B3.13 is highly virulent for mice, rapidly causing acute pulmonary and neurologic disease
Source: Nat Commun. 2025 Jul 1;16:5738. doi: 10.1038/s41467-025-60407-y (PMC12216816; doi:10.1038/s41467-025-60407-y)
Supplement: Supplementary file 7 — Transparent Peer Review file [file 41467_2025_60407_MOESM7_ESM.pdf]

nature portfolio

Peer Review File

Highly Pathogenic Avian Influenza H5N1 clade 2.3.4.4b genotype B3.13 is highly virulent for mice, rapidly causing acute and neurologic disease

Corresponding Author: Dr Heinz Feldmann

This file contains all reviewer reports in order by version, followed by all author rebuttals in order by version.

Version 0:

Reviewer comments:

Reviewer #1

(Remarks to the Author)
The work presented here compares the pathogenicity and tissue tropism of different H5N1 HPAIV, including a recent bovine H5N1 HPAIV in mice. The main result of the paper is the observation that the bovine H5N1 appeared to cause more severe disease in C57BL6 mice than other strains including the reference virulent H5N1 VN1203. This was associated with higher viral loads in the brain of mice inoculated with the bovine H5N1 than with other H5N1 strains. Given the alarming spread of bovine H5N1 and the associated risks to public health, gaining information of the biological properties of bovine H5N1 is thus very important. The experiments presented here are basic, and may need to be complemented by other experiments to provide stronger support to the conclusions.

In particular, why was a single dose of virus used. This work has to be compared with the work recently published by Eisfeld, A. J. et al. (cited as ref 23 in the discussion of the manuscript). As in the manuscript reviewed here, Eisfeld, A. J. et al. compare a bovine H5N1 with the mink H5N1 and with H5N1 VN1203. The work by Eisfeld is more complete, as infections in mice were performed with a broader range of inocula, enabling them to calculate MLD50. Importantly, the conclusions from the two manuscripts are different: Eisfeld, A. J. et al. did not find evidence for increased pathogenicity of the bovine H5N1 compared to H5N1 VN1203 or to the mink H5N1. The reasons for these discrepancies are unclear. To verify the reproducibility of the observations, the authors may verify key findings using different inocula doses, in addition to 10^5 TCID50. This is especially true for the experiments in BALBc mice, which died very quickly when inoculated with 10^5 TCID50. With this is very high dose, the viruses cause massive lung damage leaving not enough time, as the authors suggested, to allow the virus to spread systemically.

The authors describe the immune response to the different viruses in the lung and brain of infected mice. These analyses lack essential basic controls, which are non-infected mice. Indeed, without these controls, it is impossible to know what is the level of cytokine upregulation. In addition, it is unclear why the authors do not present type I IFN levels or ISG gene expression levels, which are critical parameters in virus-host interaction.

Finally, the histological lesions are described extensively, and may be shortened to present main findings more concisely.

In conclusion, the work presented here presents interesting findings, which however should be complemented by other analyses, especially to explore the discrepancies with the conclusions from Eisfeld, A. J. et al.

Minor comments
A/Vietnam/1203/2004 (H5N1) (VN1203), a reference HPAI A(H5N1) clade 2.3.4.4b virus
Wrong: this is a clade 1 H5 virus

Reviewer #2

(Remarks to the Author)

Tipih et al, Recent bovine HPAI H5N1 isolate is highly virulaent for mice, rapidly causing acute pulmonary and neurologic disease

Summary

In this manuscript, the authors have compared pathogenicity of three clade 2.3.4.4b viruses (one derived from a lactating cow during the current outbreak in the US and two earlier isolates from mink or mountain lion) and an older HPAI H5N1 isolate (VN1203) in mice. In one experiment, they orally or intranasally inoculated C57BL/6 mice with one dose of virus (105 TCID50) and (1) assessed weight loss and survival, and (2) collected tissues for virus titration, histopathology, and cytokine analysis at 4 days post-infection. They performed a similar experiment in BALB/c mice, except that tissues were collected at 2 days post-infection. Their data suggest a modest increase in pathogenicity of the bovine isolate relative to the other three strains, with higher viral titers and higher Th1 cytokine expression in the brain, especially in C57BL/6 mice, at the timepoints that were assayed.

Specific Points

1. Only one (relatively high) virus dosage was tested to compare the pathogenicity of the selected virus strains in mice. To better compare pathogenicity of these viruses, a lethal dose 50 analysis would have been preferable (at least for one of the mouse strains). For comparison of virus titers, tissue pathology, and cytokines in tissues, a lower dosage would have been preferable.

2. All viruses examined in this work encode a multi-basic cleavage site in the HA protein, which allows for the systemic spread of HPAI H5N1 viruses in mice; notably, such spread is well-established for the VN1203 virus. The finding of a lack of (or limited) systemic spread under some conditions may be due to insufficient passage of time after infection. Therefore, an additional, later timepoint needs to be assessed to determine the differences in the ability of these viruses to spread systemically versus differences in the kinetics of their systemic spread.

3. BALB/c mice were euthanized at a different timepoint (2d post-infection, see line 192 and note that this is different from what is stated in the legends of Figures S4-S9) compared to C57BL/6 mice (4 d post-infection). The authors stated that animals were euthanized when they reached a “similar disease stage,” but do not describe their criteria for making this assessment. It’s difficult to directly compare the data from two different timepoints, because there are many possible differences at the cellular level that cannot be accounted for from the animal’s symptoms. Particularly, it’s difficult to make a conclusion about an overall lack of neurotropism in BALB/c mice when BALB/c mice tissues were collected 2 days earlier. To make a direct comparison, tissues need to be collected at the same timepoint.

4. Please provide a full list (or table) of the specific amino acid differences among the three clade 2.3.4.4b viruses used in the study. Were the virus stocks sequenced to determine whether any subpopulations emerged during preparation?

5. Could the authors define specifically what they mean by hyperactivity and how it was quantified or determined?

6. Could the authors discuss why oral inoculation was not uniformly lethal for viruses other than the cow isolate?

7. Lines 81-102: Throughout this paragraph, the authors repeatedly refer to respiratory disease in both intranasally and orally inoculated mice. Although it may be true that respiratory disease is occurring in these animals, data supporting this assumption have not yet been presented at this point in the manuscript (an observation of rapid breathing is insufficient to demonstrate an infection is respiratory). Particularly for oral inoculations, describing the disease as respiratory without discussing the supporting evidence is problematic. This paragraph should be revised to avoid making unsupported statements.

8. Could the authors discuss why the cow isolate spreads more readily to other tissues after infection by the oral route? How was the oral inoculation performed (i.e., with a pipette or an oral gavage)?

9. Lines 115-117: The conclusion is that most mice infected with viruses other than the cow isolate did not show evidence of systemic infection. However, most intranasally inoculated animals (except those inoculated with the mink virus) exhibited virus in the brain, which is consistent with systemic spread (titers are lower than that observed with the cow isolate, but this does not mean that systemic spread did not occur).

10. Figures 3 and 4:
a. Please include normal/healthy tissue for comparison.
b. Please state what parts of the brain and lung tissue are shown.
c. Both figure legends state that, “Section from immunoreactive mice with histopathologic changes shown” (lines 438-439); however, the text states, “…no significant histopathologic changes were observed in evaluated brain sections…” (line 121).

11. Lines 122-124: “Abundant nucleoprotein antigen was observed in neurons, glial cells, and occasional ependymal cells… (Fig. 3B; Fig 4B).” It’s not possible to discern the cell types based on the provided images.

12. Lines 124-125: “No virus antigen was observed in any brain cell type of mice intranasally and orally inoculated with the mink and VN1203 isolates.” Three of four mice intranasally inoculated with VN1203 exhibited virus titers in the brain. Can the authors explain why they see viral titers in the brain but no antigen staining?

13. It is difficult to compare disease severity based on histopathology analyses for oral and intranasal inoculations. The authors might consider combining Figures 3 and 4 so side-by-side comparisons can be made. If pathological lesions were scored for each condition, that would further assist comparison between the different viruses and the different inoculation routes.

14. Lines 157-160: “Virus antigen detection mimicked lesion severity as the most severe changes correlated to the most immunoreactive cell types and mice that had not [sic] histopathologic changes exhibited no viral nucleoprotein immunoreactivity.” It’s not clear where to look to make this comparison.

15. Cytokine assays lack negative (uninfected) controls. It would have been preferable to include negative controls to show that cytokines are increasing after influenza infection.

16. Figure 5: What are the fold-changes for comparisons showing a significant difference?

17. Lines 238-239: “A recent paper also did not describe neurologic disease in BALB/c mice infected with the bovine isolate supporting our result here.” In the referenced paper, high virus titers were observed in the brains of BALB/c mice infected with a bovine isolate and VN1203.

18. Lines 249-250: “The absence of histological lesions, however, was intriguing as infected mice developed neurological signs.” Only a small portion of the brain tissue is shown in Figures 3 and 4, and it is unclear what portion of the brain is represented. Did the authors examine brain stem? Could damage to other neurological tissues (e.g., spinal cord) account for the observed differences? Would lesions have been more apparent if a later timepoint had been assessed?

Minor Points
1. Line 51: It’s no clear what is meant by direct droplet transmission.

2. Lines 52-53: Please add references supporting the statement about evidence of mammal-to-mammal transmission.

3. Line 68: VN1203 is not a clade 2.3.4.4b virus. The statement should be reworded to make this clearer.

4. Figure 1: The red and brown colors used in the figure panels are similar and may be difficult to distinguish for some readers, especially in the weight loss graphs. Please consider modifying the color scheme to make the graphs easier to interpret.

5. Figure 1 legend: Since the data in the figure only cover the animals that were followed through 28 days, there is no need to mention the animals that were euthanized at 4 days post-infection.

6. Line 118 subheading: “Bovine isolate caused virus replication in brain tissue.” This has already been established in the previous section. The authors might consider revising to increase relevance to the following section.

7. Line 197: What is meant by “significant viremia”? There is no indication that statistical significance was observed (Fig. S5D or S5H).

Reviewer #3

(Remarks to the Author)
The authors present an interesting manuscript on the pathogenesis of recent HPAIV H5N1 clade 2.3.4.4b viruses in mice by using different inoculation routes such as intranasal and orally. The authors show convincing that the bovine isolate is the most pathogenic variant evident by looking at overall survival, virus titers in different tissues, pathology and the potential for neuroinvasion and increase in cytokines in lung and brain tissue. Overall, the study is nicely designed, the manuscript is clearly written and the results are supported by the conclusion drawn.

Comments:
Line 48: “The human cases have thus far been mild, reporting mostly conjunctivitis although respiratory symptoms have been described in some cases 7,8. All cases have been reported in persons working at dairy and poultry farms where cows or domesticated birds tested positive for HPAI A(H5N1) suggesting direct droplet cow/poultry-to-human transmission of the virus or fomite transmission through contaminated equipment.”

I would suggest the authors to cite additionally also appropriately the recent publications regarding dairy farm worker infections in the USA. For example:
DOI: 10.15585/mmwr.mm7321e1
DOI: 10.1056/NEJMc2407264
DOI: 10.1056/NEJMc2405371

Line 57: “Prior to occurrence of HPAI A(H5N1) clade 2.3.4.4b infections in dairy cattle, outbreaks of HPAI A(H5N1) clade 2.3.4.4b viruses had been reported in mammals including farmed mink in Spain in 2022.”
I would advise the authors to be more specific in this paragraph. I assume the authors talk here about farmed-fur bearing

animals (Or about animals in farms in general) which is followed by wildlife mammals. I would advise also here to introduce the H5N1 outbreak in farmed-fur foxes in Finland (DOI: 10.2807/1560-7917.ES.2023.28.31.2300400) too.

Line 78: “Amino acid sequence comparison of all bovine segments with the corresponding segments of 3 additional isolates revealed the closest identity to the mountain lion (in most of the segments) followed by the mink with the most distantly related being the VN1203 isolate (Table S1).”
After critically reading the manuscript, I would advise the authors to optimize and look more in depth into the part on the amino acid sequence differences among the used strains. The manuscript shows differences of the recent H5N1 strains in their pathology. For example, the mink isolate did not show neuroinvasive potential in the intranasally inoculated C57BL/6 mice but showed at least low virus titers in the brain in mice which were orally inoculated. I do agree that differences in the neuroinvasive potential can have multiple reasons, meaning either viral factors and host factors. Ideally, the authors add an additional table with highlighting differences in amino acids at least in the recent H5N1 2344b clade strains. Adding this layer of information, I think adds an important foundation and maybe gives first clues for understanding the differences in neuroinvasive potential among HPAI H5N1 viruses. I do realize that even with adding this the authors cannot solve the problem, but it might help researchers in the future.

Even though the manuscript is easy understandable I would wish for an additional table regarding the respiratory and neurological pathology. There seems to be quite some variations within and among the inoculation route and it would be nice to have the information altogether maybe in a supplemental table. While reading it very carefully not all the information was easy to grab since mice strains and inoculation methods and viruses are used.

The authors have a very nice and detailed description of the overall neurological and respiratory pathology. Kudos to that! However when looking at the Figure 3 and Figure 4, even with a trained pathological point of view it is very difficult to follow their explanations. For example: In the cluster of NP positive cells within I assume the cerebral cortex of the mouse brain, I cannot decipher whether neurons, microglia and astrocytes are NP positive. It would help if the authors could provide higher magnification images so I could also evaluate whether their interpretation of the results is accurate. Looking at the H&E slides of the lungs is somewhat okay to follow their interpretation but especially in the NP IHC also here I barely can follow their interpretation, especially their interpretation of infected cell types. If I see correctly, the authors say that type-I and type-II pneumocytes are infected, however, I barely can see the alveoli in these pictures. Also here additional higher magnification pictures would be advisable for correct interpretation.

Line 190: “All mice inoculated intranasally with the different isolates succumbed to infection except for a single mouse in the mink group (Fig. S4C).”
Out of curiosity where there any noticeable problems with inoculation of the mouse that could explain this? If so, I would add this information into the results.

Line 208: “Human cases in the United States remain mild and limited in number but continued circulation with incidental spillover into mammalian populations could confer mutations resulting in more efficient transmission and increased virulence in mammals including humans.”
Also here I would advise to cite the recently published papers from above.

Line 248: “High viral load and viral antigen staining in brain tissue indicated replication of the bovine isolate in the central nervous system. The absence of histological lesions, however, was intriguing as infected mice developed neurological signs. Given the lack of detectable inflammatory infiltrates and similar lack of neuronal necrosis, neuronal metabolic disturbances are deemed the most probable explanation for the described clinical signs. The proinflammatory phenotype found in brain tissue may support this notion. Necrosis and inflammation in brain tissue notably in the cerebrum has been reported before in H5N1 infected mammals 4,20 which we did not observe here.”
While I do agree with the difference aspects on how H5N1 can cause neurological aberrances without histological lesions, I would like to have here a more discrete discussion on the different aspects of the neuropathology. I am missing a discussion point more on those lines that it is not a novel concept that influenza A viruses develop neurological complications during the acute phase of infection which can be even lasting into the subacute phase as shown in these papers DOI: 10.3389/fncel.2021.643650, DOI: 10.1523/JNEUROSCI.1740-17.2018. Additionally, what also gets more attention especially since the SARS-CoV-2 pandemic and studying the neuropathology thereof is that especially activation of astrocytes and microglia (which we know are activated at least in seasonal IAV infected mice) can contribute to the development of “mild” neurological clinical symptoms. The activation of astrocytes and microglia evident by more pronounced GFAP (Astrocyte specific staining) and IBA1 (microglia specific activation) staining along with cell body ramification are an upcoming aspect in the neuropathology especially when histopathologic evidence for inflammations (being mostly recruitment of immune cells) are absent.

Version 1:

Reviewer comments:

Reviewer #1

(Remarks to the Author)
The authors provide a revised version of the manuscript that includes useful controls, such as the inclusion of immune

response levels in mock-infected mice for comparison. They chose not to present a comparison of the pathogenicity of selected viruses at lower doses than 105 TCID50, which would have been very informative and was recommended in review 1, but rather chose to perform infections with four doses of the bovine isolate in two strains of mice.

The abstract states “the recent bovine isolate possesses enhanced respiratory and neuroinvasive/neurovirulent properties”. It is not clear what the authors mean by increased “respiratory properties”: there are no statistically significant differences in viral load, maybe the authors refer to something else and could rewrite to make it clearer?

The tile could be changed as “recent” has no meaning on the long term. The year of the isolate, the genotype and clade could be indicated instead.

It would have been useful to discuss the results with reference to Eisfled et al 2024 more extensively, discussing possible reasons for the increased pathogenicity of the bovine isolate used here compared to VN1203, versus no increase pathogenicity of the bovine isolate tested by Eisfeld compared to VN1203. This should be discussed in the discussion.

Minor: Figure S5: Change legend, as it is not only intranasal

Reviewer #2

(Remarks to the Author)
I appreciate the authors' considerations of my comments, and I am satisfied with their responses.

Reviewer #3

(Remarks to the Author)
The reviews were addressed appropriately and I advise to publish the study.

Open Access This Peer Review File is licensed under a Creative Commons Attribution 4.0 International License, which permits use, sharing, adaptation, distribution and reproduction in any medium or format, as long as you give appropriate credit to the original author(s) and the source, provide a link to the Creative Commons license, and indicate if changes were made.
In cases where reviewers are anonymous, credit should be given to 'Anonymous Referee' and the source.
The images or other third party material in this Peer Review File are included in the article’s Creative Commons license, unless indicated otherwise in a credit line to the material. If material is not included in the article’s Creative Commons license and your intended use is not permitted by statutory regulation or exceeds the permitted use, you will need to obtain permission directly from the copyright holder.
To view a copy of this license, visit https://creativecommons.org/licenses/by/4.0/

REVIEWER COMMENTS

Reviewer #1 (Remarks to the Author):

The work presented here compares the pathogenicity and tissue tropism of different H5N1 HPAIV, including a recent bovine H5N1 HPAIV in mice. The main result of the paper is the observation that the bovine H5N1 appeared to cause more severe disease in C57BL6 mice than other strains including the reference virulent H5N1 VN1203. This was associated with higher viral loads in the brain of mice inoculated with the bovine H5N1 than with other H5N1 strains. Given the alarming spread of bovine H5N1 and the associated risks to public health, gaining information of the biological properties of bovine H5N1 is thus very important. The experiments presented here are basic, and may need to be complemented by other experiments to provide stronger support to the conclusions.

In particular, why was a single dose of virus used. This work has to be compared with the work recently published by Eisfeld, A. J. et al. (cited as ref 23 in the discussion of the manuscript). As in the manuscript reviewed here, Eisfeld, A. J. et al. compare a bovine H5N1 with the mink H5N1 and with H5N1 VN1203. The work by Eisfeld is more complete, as infections in mice were performed with a broader range of inocula, enabling them to calculate MLD50. Importantly, the conclusions from the two manuscripts are different: Eisfeld, A. J. et al. did not find evidence for increased pathogenicity of the bovine H5N1 compared to H5N1 VN1203 or to the mink H5N1. The reasons for these discrepancies are unclear. To verify the reproducibility of the observations, the authors may verify key findings using different inocula doses, in addition to 10^5 TCID50. This is especially true for the experiments in BALBc mice, which died very quickly when inoculated with 10^5 TCID50. With this is very high dose, the viruses cause massive lung damage leaving not enough time, as the authors suggested, to allow the virus to spread systemically.

There are several clear differences between our study and the one by Eisfeld et al. aside from what appears to be the key sticking point, the infectious dose administered. For instance, the bovine isolates used in each study differed), the volumes of inoculum differed, and the technical delivery of the inoculum for the oral route differed . The New Mexico, Kansas and Ohio H5N1 isolates belong to the same clade but may exhibit distinct biological characteristics.

The survival studies by Eisfeld et al was performed with the H5N1 Kansas isolate for oral and with H5N1 New Mexico for intranasal route. No comparison in mortality was performed between the bovine (H5N1 Kansas or H5N1 New Mexico) isolates and either the H5N1 mink or H5N1 VN1203. In fact they compared tissue tropism on days 3 and 6 following intranasal inoculation with New Mexico H5N1, VN1203 H5N1 or Isumi-H1N1). Whereas our study directly compares pathology, survival and symptomology for 4 different H5N1 HPAI virus isolates using 2 routes of infection in 2 different strains of mice.

Eisfeld et al presented day 3 and day 6 time points for viral titers. Our day 2 data is comparable to Eisfeld day 3 data.

Contrary to the reviewer’s statements, we believe our data does not contradict the conclusions of Eisfeld et al, but rather complements the findings described by Eisfeld et al. Our data also shows similar viral titers (at early time points) in several tissues between the viral isolates but as we go on to compare survival, we do see a difference in pathology and survival. Had Eisfeld et al. made similar comparisons they may very well have had similar findings.

Nevertheless, we repeated the study using a lower dose of the bovine isolate comparing disease progression in BALB/c and C57BL/6J mice.

The authors describe the immune response to the different viruses in the lung and brain

of infected mice. These analyses lack essential basic controls, which are non-infected mice. Indeed, without these controls, it is impossible to know what is the level of cytokine upregulation. In addition, it is unclear why the authors do not present type I IFN levels or ISG gene expression levels, which are critical parameters in virus-host interaction.

As suggested by the reviewer, cytokine data has been updated to include mock infected animals. This has been added to the text. Lines 156-169, 188-191

Unfortunately, we have no material left to evaluate ISG expression levels.

Finally, the histological lesions are described extensively and may be shortened to present main findings more concisely.

The section has been shortened. Lines 128-155

In conclusion, the work presented here presents interesting findings, which however should be complemented by other analyses, especially to explore the discrepancies with the conclusions from Eisfeld, A. J. et al.

We thank the reviewer for the positive and supportive conclusion statement.

Minor comments
A/Vietnam/1203/2004 (H5N1) (VN1203), a reference HPAI A(H5N1) clade 2.3.4.4b virus
Wrong: this is a clade 1 H5 virus
Corrected in text

Reviewer #2 (Remarks to the Author):

Tipih et al, Recent bovine HPAI H5N1 isolate is highly virulaent for mice, rapidly causing acute pulmonary and neurologic disease

Summary

In this manuscript, the authors have compared pathogenicity of three clade 2.3.4.4b viruses (one derived from a lactating cow during the current outbreak in the US and two earlier isolates from mink or mountain lion) and an older HPAI H5N1 isolate (VN1203) in mice. In one experiment, they orally or intranasally inoculated C57BL/6 mice with one dose of virus (105 TCID50) and (1) assessed weight loss and survival, and (2) collected tissues for virus titration, histopathology, and cytokine analysis at 4 days post-infection. They performed a similar experiment in BALB/c mice, except that tissues were collected

at 2 days post-infection. Their data suggest a modest increase in pathogenicity of the bovine isolate relative to the other three strains, with higher viral titers and higher Th1 cytokine expression in the brain, especially in C57BL/6 mice, at the timepoints that were assayed.

Specific Points

1. Only one (relatively high) virus dosage was tested to compare the pathogenicity of the selected virus strains in mice. To better compare pathogenicity of these viruses, a lethal dose 50 analysis would have been preferable (at least for one of the mouse strains). For comparison of virus titers, tissue pathology, and cytokines in tissues, a lower dosage would have been preferable.

As recommended, we performed an LD_{50} in BALB/C and C57BL/6J mice with the H5N1 Ohio isolate using the intranasal exposure route. We obtained LD_{50}'s of 1.8 $TCID_{50}$ and 3.2 $TCID_{50}$ for BALB/c and C57BL/6J mice, respectively. Since the bovine isolate is of highest public health significance and compares to the isolates studied by Eisfeld et al, we repeated experiments using a 1,000 LD50 (10^3 $TCID_{50}$) of the bovine isolate in BALB/c and C57BL/6J mice. Results have been added to the manuscript.

2. All viruses examined in this work encode a multi-basic cleavage site in the HA protein, which allows for the systemic spread of HPAI H5N1 viruses in mice; notably, such spread is well-established for the VN1203 virus. The finding of a lack of (or limited) systemic spread under some conditions may be due to insufficient passage of time after infection. Therefore, an additional, later timepoint needs to be assessed to determine the differences in the ability of these viruses to spread systemically versus differences in the kinetics of their systemic spread.

More time points assessing virus replication are always better, but a study needs to remain feasible and animal numbers ethically justified. Virus isolates are the same or very similar in passage number and the infectious dose is the same for each isolate allowing a direct comparison. If an isolate needs more replication rounds to achieve the same than other isolates it means that replication kinetics are reduced. With an early time point for virus replication and survival, one should be able to achieve the goals to compare virus replication and infection outcome. This approach is commonly used in many studies. In addition, the studies described here were greatly influenced by the appearance of severe disease and the quick time to death in the bovine infected animals. Particularly in the BALB/c mice where animals were meeting euthanasia criteria at (and sometimes before) our scheduled time points. If the virus has not spread systemically at these late time points (severe disease) we are confident there will not be systemic infection. Nevertheless, we have added an additional harvesting time point for the low dose challenge with the bovine isolate.

3. BALB/c mice were euthanized at a different timepoint (2d post-infection, see line 192 and note that this is different from what is stated in the legends of Figures S4-S9) compared to C57BL/6 mice (4 d post-infection). The authors stated that animals were euthanized when they reached a “similar disease stage,” but do not describe their criteria for making this assessment. It’s difficult to directly compare the data from two different timepoints, because there are many possible differences at the cellular level that cannot be accounted for from the animal’s symptoms. Particularly, it’s difficult to make a conclusion about an overall lack of neurotropism in BALB/c mice when BALB/c mice tissues were collected 2 days earlier. To make a direct comparison, tissues need to be collected at the same timepoint.

BALB/c mice were euthanized at day 2 post-inoculation. This has been corrected in the figure legends. Animals were euthanized when they began to show clinical signs (weight loss, ruffled fur or reduced activity).

With the differences in disease kinetics it was impossible to collect the same timepoints as the BALB/c mice succumbed to infection at D3, meaning there were no mice to sample at D4. Therefore, the study was designed to measure parameters at the similar disease points rather than the same timepoint after infection. This allows us to compare viral replication and pathology at a disease state (severe), rather than a point in time. However, a second study has now been run with a lower infectious dose and samples were collected at both D2 and D4 post-inoculation for comparison.

4. Please provide a full list (or table) of the specific amino acid differences among the three clade 2.3.4.4b viruses used in the study. Were the virus stocks sequenced to determine whether any subpopulations emerged during preparation?

Virus stocks were sequenced, and amino acid differences (consensus sequence) were provided (Table S1-S9)

5. Could the authors define specifically what they mean by hyperactivity and how it was quantified or determined?

Hyperactivity was defined as excessive movement upon observation. In this case, mice would circle the cage repeatedly without stimulation or rest. It was not quantified but repeated and consistent observation was a determining factor.

6. Could the authors discuss why oral inoculation was not uniformly lethal for viruses other than the cow isolate?

We can do nothing but speculate on this and this was addressed in the discussion. (Lines 305-312). It is also unclear why lethality after orogastric inoculation was observed

only with the bovine isolate. Amino acid comparison shows that the mink, mountain lion and VN1203 isolates harbor the 497K in the polymerase acidic (PA) protein. Mutant H7N9 viruses possessing the 497K displayed significantly lower lung viral titers and increased LD_{50} in mice compared to wild type viruses suggesting that the amino acid change decreased polymerase activity and virulence. Future studies need to address the role of sequence differences to understand and define molecular signatures that explain the increased virulence and altered organ tropism of the bovine isolate in mammalian species.

7. Lines 81-102: Throughout this paragraph, the authors repeatedly refer to respiratory disease in both intranasally and orally inoculated mice. Although it may be true that respiratory disease is occurring in these animals, data supporting this assumption have not yet been presented at this point in the manuscript (an observation of rapid breathing is insufficient to demonstrate an infection is respiratory). Particularly for oral inoculations, describing the disease as respiratory without discussing the supporting evidence is problematic. This paragraph should be revised to avoid making unsupported statements.

The paragraph has been revised. Lines 91-112

8. Could the authors discuss why the cow isolate spreads more readily to other tissues after infection by the oral route? How was the oral inoculation performed (i.e., with a pipette or an oral gavage)?

We refer the reviewer to our response to comment number 6

The following has been added to text. Line 355-356

"For orogastric inoculation, inoculum was deposited into the stomach using a feeding tube"

9. Lines 115-117: The conclusion is that most mice infected with viruses other than the cow isolate did not show evidence of systemic infection. However, most intranasally inoculated animals (except those inoculated with the mink virus) exhibited virus in the brain, which is consistent with systemic spread (titers are lower than that observed with the cow isolate, but this does not mean that systemic spread did not occur).

The sentence has been modified to read. Lines 125-127 "In contrast, most of the mice inoculated with the mink, mountain lion, and VN1203 isolates manifested limited evidence of systemic infection". For intranasal infection, virus can enter the brain through the olfactory route and doesn't necessarily need systemic spread.

10. Figures 3 and 4:
a. Please include normal/healthy tissue for comparison.

Tissue from mock infected animals are provided for comparison

b. Please state what parts of the brain and lung tissue are shown.

Hemi-sections of skull and insufflated lung were taken for histopathologic evaluation.

c. Both figure legends state that, “Section from immunoreactive mice with histopathologic changes shown” (lines 438-439); however, the text states, “…no significant histopathologic changes were observed in evaluated brain sections…” (line 121).

“Section from immunoreactive mice with histopathologic changes shown” has been modified to read “Representative sections from each group shown”.

11. Lines 122-124: “Abundant nucleoprotein antigen was observed in neurons, glial cells, and occasional ependymal cells… (Fig. 3B; Fig 4B).” It’s not possible to discern the cell types based on the provided images.

High magnification images provided (Fig S1)

12. Lines 124-125: “No virus antigen was observed in any brain cell type of mice intranasally and orally inoculated with the mink and VN1203 isolates.” Three of four mice intranasally inoculated with VN1203 exhibited virus titers in the brain. Can the authors explain why they see viral titers in the brain but no antigen staining?

The reviewer does bring up the limitations of different techniques and how these can lead to inconsistencies. In this study, the brain was divided in half by separating the hemispheres. One hemisphere was immediately put into fixative (formalin) for histology and the other was used for viral loads, infectious titers (and cytokine analysis depending on the experiment). The infectious titers displayed were determined by using a sample of an entire hemisphere of brain while the IHC is one very small slice of brain (approximately 2uM thick). So it’s a matter of chance if we detect IHC in the brain if replication is low. Combined with the fact that antigen detection is less sensitive than infectious assays. There isn’t enough replicating virus to reliably or consistently detect viral antigen.

Immunohistochemistry is less sensitive compared to the $TCID_{50}$ assay

13. It is difficult to compare disease severity based on histopathology analyses for oral and intranasal inoculations. The authors might consider combining Figures 3 and 4 so side-by-side comparisons can be made. If pathological lesions were scored for each

condition, that would further assist comparison between the different viruses and the different inoculation routes.

Table S10-12 comparing orogastric and intranasal histological scores for brain and lung pathology has been added.

14. Lines 157-160: “Virus antigen detection mimicked lesion severity as the most severe changes correlated to the most immunoreactive cell types and mice that had not [sic] histopathologic changes exhibited no viral nucleoprotein immunoreactivity.” It’s not clear where to look to make this comparison.

Lines 128-155. The histopathology section has been shortened to reflect the key findings.

15. Cytokine assays lack negative (uninfected) controls. It would have been preferable to include negative controls to show that cytokines are increasing after influenza infection.

Cytokine assays were repeated with uninfected controls.

16. Figure 5: What are the fold-changes for comparisons showing a significant difference?

We are not sure what the reviewer is asking for. However, cytokine levels in lung homogenate filtrate were determined using the Bio-Plex Pro^{TM} Mouse Cytokine 23-plex immunoassay that utilizes magnetic bead-based technology to capture and detect the target analytes. Differences in cytokine induction by the H5N1 viruses were performed using one-way ANOVA with Tukey’s multiple comparison.

17. Lines 238-239: “A recent paper also did not describe neurologic disease in BALB/c mice infected with the bovine isolate supporting our result here.” In the referenced paper, high virus titers were observed in the brains of BALB/c mice infected with a bovine isolate and VN1203.

Lines 266-268. The sentence has been modified to read. “Furthermore, neurotropism of a bovine HPAI A(H5N1) clade2.3.4.4b isolate in BALB/c mice was recently reported without mentioning of neurologic disease signs”.

18. Lines 249-250: “The absence of histological lesions, however, was intriguing as infected mice developed neurological signs.” Only a small portion of the brain tissue is shown in Figures 3 and 4, and it is unclear what portion of the brain is represented. Did the authors examine brain stem? Could damage to other neurological tissues (e.g., spinal cord) account for the observed differences? Would lesions have been more apparent if a later timepoint had been assessed?

Hemi-sections of the brain were analyzed for histopathology. We still did not observe lesions with a lower inoculation dose in our follow up study. Its possible lesions can be observed at a later time point but in our experiments animals succumb starting at day 4 and for comparison purposes our latest time point is day 4.

Minor Points

1. Line 51: It's no clear what is meant by direct droplet transmission.

The introduction has been modified to include latest data on human case count and sources of viral exposure.

Line 55-56. The phrase "direct droplet cow/poultry-to-human transmission of the virus or fomite transmission through contaminated equipment" has been replaced with "occupational exposure"

2. Lines 52-53: Please add references supporting the statement about evidence of mammal-to-mammal transmission.

The following references have been added.

DOI: 10.1056/NEJMc2407264
DOI: 10.1056/NEJMc2405371

3. Line 68: VN1203 is not a clade 2.3.4.4b virus. The statement should be reworded to make this clearer.

The sentence has been revised to read "In this study, we compared the virulence of A/Vietnam/1203/2004 (H5N1) (VN1203), a reference HPAI A(H5N1) clade 1 virus, to three recent HPAI A(H5N1) clade 2.3.4.4b virus isolates (A/bovine/OH/B24OSU-342/2024 (bovine), A/mountain lion/MT/1/2024 (mountain lion), and A/mink/Spain/3691-8_22VIR10586-10/2022 (mink) described above in mice inoculated with a high dose (10^5 $TCID_{50}$) of each of the HPAI A(H5N1) virus isolates". (Lines 73-77)

4. Figure 1: The red and brown colors used in the figure panels are similar and may be difficult to distinguish for some readers, especially in the weight loss graphs. Please consider modifying the color scheme to make the graphs easier to interpret.
The color scheme has been modified

5. Figure 1 legend: Since the data in the figure only cover the animals that were followed through 28 days, there is no need to mention the animals that were euthanized at 4 days post-infection.

The legend has been modified to only include animals that were monitored for survival.

6. Line 118 subheading: "Bovine isolate caused virus replication in brain tissue." This

has already been established in the previous section. The authors might consider revising to increase relevance to the following section.

Subheading has been updated to read "Bovine isolate caused neurologic disease in the absence of histologic lesions" Line 128

7. Line 197: What is meant by "significant viremia"? There is no indication that statistical significance was observed (Fig. S5D or S5H).

The sentence has been modified to read "infectious virus was found in blood samples of 3/4 intranasally and in 4/4 orogastric infected BALB/c mice". Line 187-188

Reviewer #3 (Remarks to the Author):

The authors present an interesting manuscript on the pathogenesis of recent HPAIV H5N1 clade 2.3.4.4b viruses in mice by using different inoculation routes such as intranasal and orally. The authors show convincing that the bovine isolate is the most pathogenic variant evident by looking at overall survival, virus titers in different tissues, pathology and the potential for neuroinvasion and increase in cytokines in lung and brain tissue. Overall, the study is nicely designed, the manuscript is clearly written and the results are supported by the conclusion drawn.

Comments:
Line 48: "The human cases have thus far been mild, reporting mostly conjunctivitis although
respiratory symptoms have been described in some cases 7,8. All cases have been reported in persons working at dairy and poultry farms where cows or domesticated birds tested positive for HPAI A(H5N1) suggesting direct droplet cow/poultry-to-human transmission of the virus or fomite transmission through contaminated equipment."

I would suggest the authors to cite additionally also appropriately the recent publications regarding dairy farm worker infections in the USA. For example:
DOI: 10.15585/mmwr.mm7321e1
DOI: 10.1056/NEJMc2407264
DOI: 10.1056/NEJMc2405371

References have been added

Line 57: "Prior to occurrence of HPAI A(H5N1) clade 2.3.4.4b infections in dairy cattle, outbreaks of HPAI A(H5N1) clade 2.3.4.4b viruses had been reported in mammals including farmed mink in Spain in 2022."
I would advise the authors to be more specific in this paragraph. I assume the authors talk here about farmed-fur bearing animals (Or about animals in farms in general) which is followed by wildlife mammals. I would advise also here to introduce the H5N1

outbreak in farmed-fur foxes in Finland (DOI: 10.2807/1560-7917.ES.2023.28.31.2300400) too.

References have been updated

Line 78: “Amino acid sequence comparison of all bovine segments with the corresponding segments of 3 additional isolates revealed the closest identity to the mountain lion (in most of the segments) followed by the mink with the most distantly related being the VN1203 isolate (Table S1).”
After critically reading the manuscript, I would advise the authors to optimize and look more in depth into the part on the amino acid sequence differences among the used strains. The manuscript shows differences of the recent H5N1 strains in their pathology. For example, the mink isolate did not show neuroinvasive potential in the intranasally inoculated C57BL/6 mice but showed at least low virus titers in the brain in mice which were orally inoculated. I do agree that differences in the neuroinvasive potential can have multiple reasons, meaning either viral factors and host factors. Ideally, the authors add an additional table with highlighting differences in amino acids at least in the recent H5N1 2344b clade strains. Adding this layer of information, I think adds an important foundation and maybe gives first clues for understanding the differences in neuroinvasive potential among HPAI H5N1 viruses. I do realize that even with adding this the authors cannot solve the problem, but it might help researchers in the future.

Even though the manuscript is easy understandable I would wish for an additional table regarding the respiratory and neurological pathology. There seems to be quite some variations within and among the inoculation route and it would be nice to have the information altogether maybe in a supplemental table. While reading it very carefully not all the information was easy to grab since mice strains and inoculation methods and viruses are used.

The reviewer is correct, we cannot point to the cause in the differences in neuroinvasion at this time. We appreciate and agree with the suggestion of an additional table highlighting the AA differences between isolates and it has been added (Tables S1-9). We have also added a tables S10-12 comparing orogastric and intranasal histological scores for brain and lung pathology provided to help clarify those results.

The authors have a very nice and detailed description of the overall neurological and respiratory pathology. Kudos to that! However when looking at the Figure 3 and Figure 4, even with a trained pathological point of view it is very difficult to follow their explanations. For example: In the cluster of NP positive cells within I assume the cerebral cortex of the mouse brain, I cannot decipher whether neurons, microglia and astrocytes are NP positive. It would help if the authors could provide higher

magnification images so I could also evaluate whether their interpretation of the results is accurate. Looking at the H&E slides of the lungs is somewhat okay to follow their interpretation but especially in the NP IHC also here I barely can follow their interpretation, especially their interpretation of infected cell types. If I see correctly, the authors say that type-I and type-II pneumocytes are infected, however, I barely can see the alveoli in these pictures. Also here additional higher magnification pictures would be advisable for correct interpretation.

We thank the reviewer for their complement and have now added higher magnification images in the supplemental material for better clarity of the observed lung pathology. (Fig S1)

Line 190: "All mice inoculated intranasally with the different isolates succumbed to infection except for a single mouse in the mink group (Fig. S4C)."
Out of curiosity where there any noticeable problems with inoculation of the mouse that could explain this? If so, I would add this information into the results.

We appreciate the reviewer's knowledge and understanding that not all inoculations are textbook or equal. However, in this case, we did not observe any discrepancies during inoculations in this study.

Line 208: "Human cases in the United States remain mild and limited in number but continued circulation with incidental spillover into mammalian populations could confer mutations resulting in more efficient transmission and increased virulence in mammals including humans."
Also here I would advise to cite the recently published papers from above.

We have updated and included additional references.

Line 248: "High viral load and viral antigen staining in brain tissue indicated replication of the bovine isolate in the central nervous system. The absence of histological lesions, however, was intriguing as infected mice developed neurological signs. Given the lack of detectable inflammatory infiltrates and similar lack of neuronal necrosis, neuronal metabolic disturbances are deemed the most probable explanation for the described clinical signs. The proinflammatory phenotype found in brain tissue may support this notion. Necrosis and inflammation in brain tissue notably in the cerebrum has been reported before in H5N1 infected mammals 4,20 which we did not observe here."

While I do agree with the difference aspects on how H5N1 can cause neurological aberrances without histological lesions, I would like to have here a more discrete discussion on the different aspects of the neuropathology. I am missing a discussion

point more on those lines that it is not a novel concept that influenza A viruses develop neurological complications during the acute phase of infection which can be even lasting into the subacute phase as shown in these papers DOI: 10.3389/fncel.2021.643650, DOI: 10.1523/JNEUROSCI.1740-17.2018. Additionally, what also gets more attention especially since the SARS-CoV-2 pandemic and studying the neuropathology thereof is that especially activation of astrocytes and microglia (which we know are activated at least in seasonal IAV infected mice) can contribute to the development of “mild” neurological clinical symptoms. The activation of astrocytes and microglia evident by more pronounced GFAP (Astrocyte specific staining) and IBA1 (microglia specific activation) staining along with cell body ramification are an upcoming aspect in the neuropathology especially when histopathologic evidence for inflammations (being mostly recruitment of immune cells) are absent.

We thank the reviewer for suggestions for future studies that largely mirror our own thoughts and plans. Mechanisms of neurologic disease in H5N1 infected mice was not the objective of the current study. We have started additional studies seeking to decipher the mechanisms of neuropathy in C57BL/6J mice.

REVIEWERS' COMMENTS

Dear Editor in Chief

We thank the reviewers for their insightful comments which were helpful and certainly improved our manuscript. We have provided a point-to-point response to the comments below.

Reviewer #1 (Remarks to the Author):

The authors provide a revised version of the manuscript that includes useful controls, such as the inclusion of immune response levels in mock-infected mice for comparison. They chose not to present a comparison of the pathogenicity of selected viruses at lower doses than 105 TCID50, which would have been very informative and was recommended in review 1, but rather chose to perform infections with four doses of the bovine isolate in two strains of mice.

The abstract states “the recent bovine isolate possesses enhanced respiratory and neuroinvasive/neurovirulent properties”. It is not clear what the authors mean by increased “respiratory properties”: there are no statistically significant differences in viral load, maybe the authors refer to something else and could rewrite to make it clearer?

Portions of the abstract has been re-written denoting only enhanced neuroinvasive/neurovirulent disease in C57BL/6J mice.

The tile could be changed as “recent” has no meaning on the long term. The year of the isolate, the genotype and clade could be indicated instead.

The title has been changed to:

Highly Pathogenic Avian Influenza H5N1 clade 2.3.4.4b genotype B3.13 is highly virulent for mice, rapidly causing acute and neurologic disease.

It would have been useful to discuss the results with reference to Eisfled et al 2024 more extensively, discussing possible reasons for the increased pathogenicity of the bovine isolate used here compared to VN1203, versus no increase pathogenicity of the bovine isolate tested by Eisfeld compared to VN1203. This should be discussed in the discussion.

A paragraph has been added to address the comment. Lines 308-315

Minor: Figure S5: Change legend, as it is not only intranasal

The legend has been updated to indicate intranasal and orogastric inoculations.

Reviewer #2 (Remarks to the Author):

I appreciate the authors' considerations of my comments, and I am satisfied with their responses.

Reviewer #3 (Remarks to the Author):

The reviews were addressed appropriately and I advise to publish the study.
